# Genetically Encoding Stimulated Raman-Scattering Probes for Cell Imaging Using Infrared Fluorescent Proteins

**DOI:** 10.1021/acs.jpclett.6c02085

**Published:** 2026-08-11

**Authors:** David Regan, Ozan Aksakal, John J.K. McLarnon, Athena Zitti, Magdalena Lipka-Lloyd, Pierre J. Rizkallah, Anna J. Warren, Peter D. Watson, Wolfgang Langbein, D. Dafydd Jones, Paola Borri

**Affiliations:** † School of Biosciences, 2112 Cardiff University, Cardiff CF10 3AX, U.K.; ‡ School of Medicine, Cardiff University, Cardiff CF14 4XN, U.K.; § Diamond Light Source, Ltd., Harwell Science and Innovation Campus, Didcot OX11 0DE, U.K.; ∥ School of Physics and Astronomy, Cardiff University, Cardiff CF24 3AA, U.K.

## Abstract

Stimulated Raman scattering (SRS) microscopy offers great potential to surpass fluorescent-based approaches, owing to the sharp line width of Raman vibrations amenable to supermultiplex cell imaging, but currently lacks one crucial component: genetically encodable tags equivalent to fluorescent proteins. Here, we show that infrared fluorescent proteins (IRFPs) can be used as genetically encoded SRS probes and benefit from the electronic preresonant SRS enhancement effect with near-infrared exciting pulses, comparable to synthetic dyes reported in the literature. SRS imaging of the nucleus in mammalian cells is demonstrated where a histone protein is fused to an IRFP. This work opens the route toward Raman-based cell imaging using genetically encoded probes, motivating efforts in solving the challenges of photostability and creating a vibrational palette.

## Graphical abstract

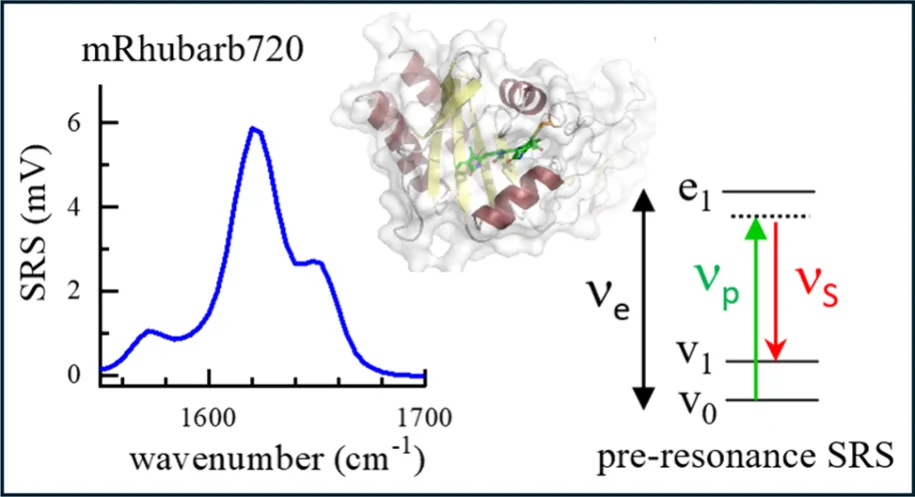

The ability to study complex biomolecular events *in situ* using optical microscopy has revolutionized our understanding of fundamental processes essential to life. The underlying paradigm shift was coupling fluorescence microscopy with probes such as the green fluorescent protein (GFP)^1–3^ that allowed generation of genetic fusions, whereby only specific molecular targets are labeled directly within the cell. Beyond GFP, many fluorescent proteins (FPs) spanning the near-ultraviolet, visible and near-infrared regions have been developed, expanding the utility of fluorescence microscopy. These include infrared fluorescent proteins, such as mRhubarb720,^4^ emIRFP670,^5^ and mIRFP670nano3,^6^ that use the haem breakdown product biliverdin Xi-α (BV) as the fluorescent cofactor (Figure 1a). Far-red FPs such as mCherry^7,8^ have the chromophore directly encoded within the amino acid sequence and formed through a post-translational covalent rearrangement event (Figure 1b).

**Figure 1:**
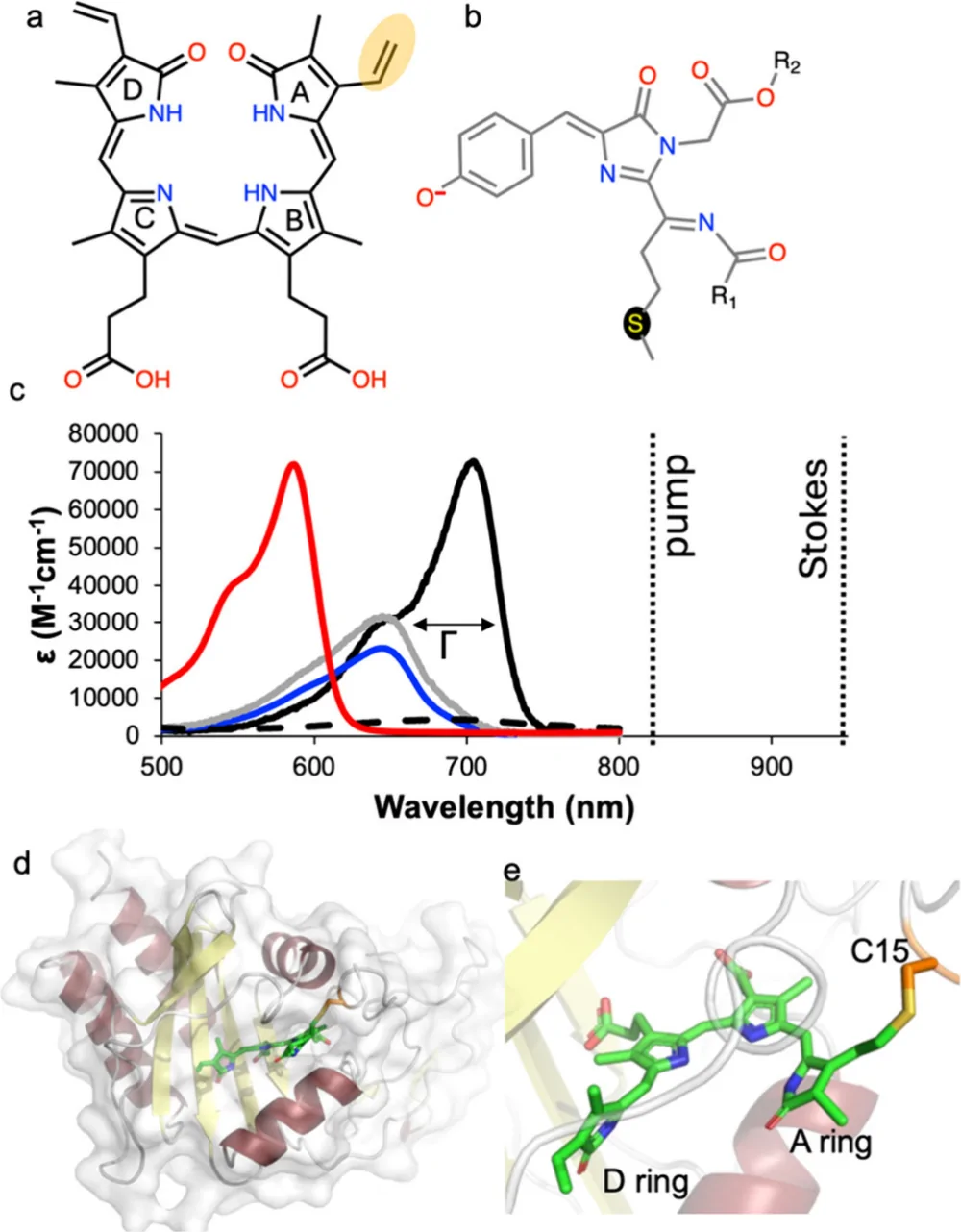
Molecular and spectral features of IRFPs. (a) The biliverdin chromophore common to the investigated IRFPs. The C=C bond highlighted in orange forms a thioether link to the protein component. (b) The chromophore structure of mCherry. (c) Measured absorbance spectra of mRhubarb720 (black solid), mIRFP670nano3 (gray), emiRFP670 (blue), mCherry (red), and free biliverdin (black dashed). Γ is the line width of the electronic transition. The pump and Stokes laser wavelengths in the SRS setup are shown as dotted lines. (d) The overall molecular structure of mRhubarb determined here, with (e) a close-up of biliverdin (green sticks).

Fluorescence microscopy using FPs is the current method of choice for cell imaging, however some limitations remain. Organic fluorophores are prone to photobleaching, limiting time course observations and quantitative analysis, and can be cytotoxic.^9–12^ IRFPs also have poor quantum yields (<15%).^5,13^ Moreover, fluorescence excitation and emission spectra are intrinsically broad (typically 50 to 100 nm in wavelength or 1000 to 2000 cm^–1^ in wavenumber) and featureless. This generates a “colour barrier” that restricts the number of distinguishable fluorescent probes, and corresponding biomolecular targets, typically to about 5 probes per cell. The latter limit is especially important, as the ever-expanding complexity of biological systems requires the simultaneous monitoring of a multitude of biochemical components (often more than 20), and these are impossible to track simultaneously with current fluorescence techniques.

Vibrational microscopy based on Raman scattering provides an alternative approach.^14^ The inelastic Raman scattering of light by vibrating chemical bonds is photostable, and vibrational resonances of most biomolecules in liquids are spectrally narrow (below 20 cm^–1^). However, a major limitation of spontaneous Raman scattering is that photon fluxes in detection are low, due to the small Raman scattering cross sections of typical biomolecules. As a result, Raman microscopy requires long integration times and/or large incident powers, often incompatible with imaging living cells. Furthermore, fluorescence backgrounds can overwhelm the Raman signal. To overcome these limitations several approaches have been proposed.^15^ One of the most exciting methods, electronically preresonant coherent Raman scattering (EPR-CRS),^16^ combines the sensitivity of electronically resonant Raman scattering with the coherent enhancement of nonlinear CRS.^17,18^ Typical CRS setups use near-IR laser sources to achieve deep penetration while minimizing multiphoton damage. Hence EPR-CRS requires a far-red/near-IR chromophore, to fulfill the electronically preresonant Raman condition (Figure 1c) increasing the Raman-scattering cross-section. This concept was demonstrated experimentally with commercial near-IR absorbing small molecule dyes.^16^ However, to date, there has been limited research regarding the EPR-CRS characteristics of protein-based systems active in the infrared region,^19,20^ and to our knowledge no reports expanding their use as genetically encoded fusion tags *in situ*. Here, we show that IRFPs, and in particular mRhubarb720, provide strong EPR-CRS, and demonstrate their application in cell imaging.

For a protein to act as a genetically encoded imaging tag, it needs to be soluble, monomeric and capable of being functionally expressed in a variety of organisms; mRhubarb720 fits these criteria.^1^ In terms of electronic excitation properties, mRhubarb720 is one of the most red-shifted FPs (absorption peak wavelength λ_max_ at 703 nm) with a relatively high molar absorbance (ε = 72.5 mM^–1^ cm^–1^) compared to other IRFPs such as emIRFP670 and mIRFP670nano3 (Figure 1c and Table S1). IRFPs are qualitatively different from far-red FPs such as mCherry^7,8^ which have classical FP β-barrel structures (Figure S1) and sequence encoded chromophores that are hypsochromically shifted compared to the IFRPs (Figure 1b-c).

Using X-ray microcrystal diffraction data (resolution 3.3Å) combined with AlphaFold2 ^21,22^ modeling, we determined the structure of mRhubarb720 (Figure 1d-e and Figure S2, with Table S2 showing the structural statistics). mRhubarb720 shares a general PAS-GAF two domain structural fold with other biliverdin binding bacterial phytochrome proteins.^23,24^ In mRhubarb720, biliverdin is bound to Cys15 in the GAF domain region via a thioether link to the C3 carbon of the A ring (Figure 1a,e and Figure S2a); the D-ring occupies the 15Za isomeric form. The three main mutations around the chromophore (L196Q, T202D and V203I; see Figure S2b) are likely to give rise to red-shifted spectral characteristics compared to its predecessor iRFP713.^13^ L196Q removes a hydrophobic residue and potentially contributes to a polar side-chain interaction network with E191, K193 and Y198 that plays a role in photoconversion between the two chromophore conformational states, Pr and Pfr^4,25^ (Figure S2c). The T202D mutation results in a negatively charged carboxyl group becoming surface exposed, which is likely to improve solubility. Finally, the V203I mutation extends the interaction between the protein and the C-ring of BV (Figure S2b).

To benefit from electronic preresonant CRS, the pump (ν_p_) and Stokes (ν_S_) laser frequencies used to coherently drive molecular vibrations have to be close to the absorption maximum (ν_e_) of the chromophore, while being sufficiently red-shifted to suppress photobleaching. The Raman scattering cross-section scales with the frequency detuning^26^ as (ν_e_-ν_p_)^−4^. Since ν_p_ is closer to the electronic transition frequency than ν_S_, the resonance enhancement effect is dominated by the pump. It has been suggested in the literature by Min and colleagues^16^ that a suitable excitation frequency is detuned (red-shifted) between 2 and 6 times the homogeneous line width Γ of the electronic transition (Figure 1c). However, it has also been pointed out that the specific properties of the chromophore need to be considered to find an optimal excitation.^18,26^ An important consideration is the suppression of one-photon absorption of the pump, and in turn any associated photobleaching. In the low-energy tail of the absorption spectrum, one-photon absorption scales with the probability of finding a molecule in the electronic ground state S_0_ which is vibrationally excited by thermal occupation. With decreasing photon energy, this probability is exponentially decaying with a slope given by the thermal energy at room temperature of about 205 cm^–1^. For mRhubarb720 at 820 nm pump wavelength, having a detuning of 1790 cm^–1^ from the absorption transition without vibrational excitation, this results in a reduction of the absorption by about 4 orders of magnitude (see also Section S8). The Franck–Condon overlap provides a further factor reducing the absorption. This overlap can be estimated from the reduction of the absorption toward the blue side of the absorption peak in Figure 1c, which is dominated by the creation of vibrations in the electronic excited state S_1_ and does not require thermal occupation. A reduction by a factor around 3 for the given pump detuning can be deduced.

In addition to one-photon absorption, two-photon absorption (TPA) of the exciting pump and Stokes beams has to be considered as an electronic excitation mechanism that may lead to photobleaching. TPA is also a source of background in CRS. For example, in SRS measured as stimulated Raman loss (SRL) in the pump beam transmission, pump-Stokes TPA manifests as an apparent signal, due to the additional pump photon loss mediated by this absorption. Notably, TPA is, as CRS, a third-order nonlinear optical process. Therefore, it scales similar to CRS with the laser excitation pulse duration and power and thus cannot be suppressed relative to CRS using these parameters. Typical TPA cross-section spectra are broad, reflecting the properties of the higher electronic states of the molecules, and TPA accordingly provides a broad background in the CRS spectra. Notably, the best-performing EPR-CRS probes in terms of signal to background ratio and photostability reported so far are close to inversion symmetric, which is suppressing TPA, as discussed in Section S8 in the Supporting Information.

The SRS setup used in this study is shown in Figure S3 and operates with a fixed pump wavelength centered at 820 nm and a tunable Stokes wavelength, adjusted according to the vibrational resonance range of interest, for example at 956 nm (Figure 1c) resulting in a center wavenumber of 1740 cm^–1^. This pump wavelength is fulfilling the EPR-CRS condition for mRhubarb720 with an absorption peak wavelength λ_max_ of 703 nm. For the mIRFP670nano3, emiRFP670 and mCherry chromophores, ν_e_ is blue-shifted, hence we expect a lower EPR-CRS effect (see Figure S4). To measure SRL spectra we use spectral focusing as detailed previously^27^ and outlined in Section S5. In this approach, pump and Stokes pulses of about 100 fs duration are equally linearly chirped to about 3 ps duration, providing a spectral resolution of 11 cm^–1^. This resolution is optimal for the resonances probed as discussed in Section S5 and shown in Figure S5.

By changing the delay time between the pulses, the instantaneous frequency difference (IFD) is tuned, allowing to record an SRL spectrum, as shown in Figure 2a (dotted line) for mRhubarb720. As mentioned, when pump and Stokes pulses are in temporal overlap, a combined two-photon absorption process occurs (namely the absorption of ν_p_+ν_S_, see Figure S6 and S7) which manifests as a pump-loss and thus appears as SRL signal in our configuration. This TPA pump-Stokes cross-correlation profile has subsequently been subtracted from the data, to highlight the vibrational SRL contribution, and the corresponding spectra are shown in Figure 2 as solid lines (see Figure S8 and S9 for all measured spectra before subtraction and Section S5 for procedural details). Vibrational resonances around 1620–1650 cm^–1^ are observed, characteristic of the C=C and C=N bonds which dominate the biliverdin chromophore (Figure 1a). When compared with predicted spectral characteristics, the peaks around 1620 cm^–1^ and 1650 cm^–1^ are likely to correspond to C=C stretches in the B-/C-rings and the A-/D-rings, respectively (Section S6, Figure S13). Notably, when tuning the Stokes wavelength to a center wavenumber of 1640 cm^–1^ (blue line Figure 2a), these resonances are more efficiently driven as compared to using 1740 cm^–1^ (black line Figure 2a). This is because the center wavenumber corresponds to the maximum temporal overlap between pump and Stokes (see also Figure S8 and S10). Hence, when the center is tuned near the vibrational resonance of interest, an optimal use of the excitation intensity pulse overlap is obtained. Indeed, considering the spectra for mRhubarb720 in Figure 2a, we see a 7-fold increase in the SRL signal for the 1620 cm^–1^ resonance when using 1640 cm^–1^ as the center IFD compared to 1740 cm^–1^. We verified that the SRL signal scales linearly with the pump power, and that the signal disappears when blocking one of the beams, as expected (Figure S6). For comparison, we measured the SRL spectrum of free BV in solution (Figure 2a), i.e. not bound to an IRFP. Interestingly, we found a spectrum lacking significant vibrational resonances, in keeping with the predicted spectral properties (Figure S13). We attribute this difference to the solvation environment of free BV, as compared to BV bound to the interior of the protein component, resulting in a much weaker, broader and shifted absorption spectrum (see Figure 1c and S11a), suppressing the EPR enhancement of SRS. The comparison of the SRL spectra in Figure S11b shows a hundredfold reduction of the signal from free BV and a vibrational response broadened and shifted to 1550–1620 cm^–1^. Simply put, binding of BV to the protein provides a stable environment and maintains BV in a defined conformation, allowing for enhanced absorption and longer vibrational coherence. Importantly, the lack of significant SRL from free BV enables detecting IRFPs with negligible SRL background from any unbound cofactor naturally present in cells.

**Figure 2:**
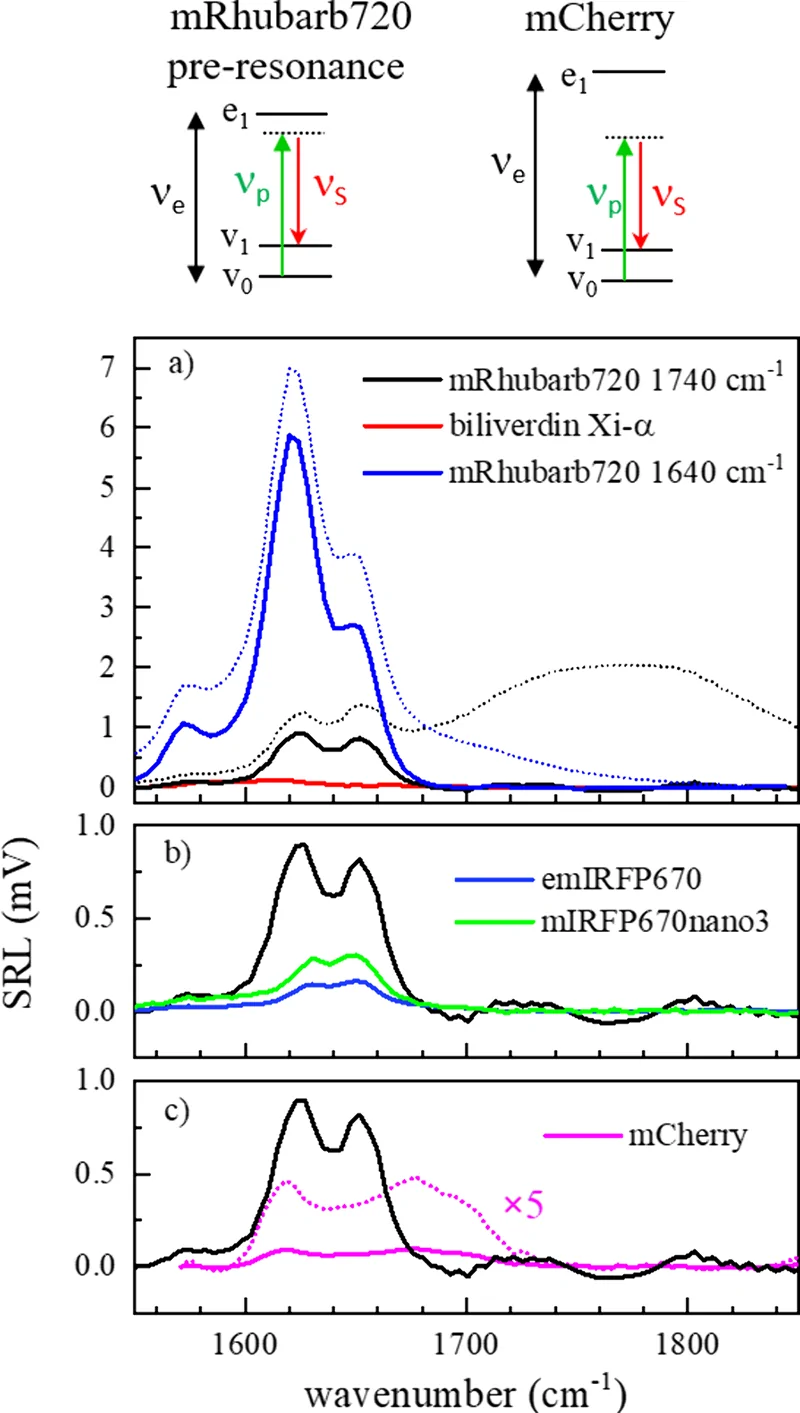
Stimulated Raman loss spectra of IRFPs in solution (1 mM concentration in 50 mM Tris buffer, 1740 cm^–1^ center IFD). (a) BV with 50 mM NaOH (red), mRhubarb720 (black), also at 1640 cm^–1^ center IFD (blue); dotted lines as measured, solid lines TPA subtracted. (b) BV-containing IRFPs mRhubarb720 (black), mIRFP670nano3 (green) and emIRFP670 (blue). (c) mCherry (magenta, dotted line scaled by factor 5) compared to mRhubarb720 (black). Top: diagrams illustrating the energy separation between the electronic transition and the pump frequency in our SRL setup, for mRhubarb720 and mCherry. ν_e_ is the electronically resonant absorption frequency of the chromophore, v_0_ and v_1_ represent the ground and excited vibrational states, respectively, whereas e_1_ is the first excited electronic state. Microscope objective 20× 0.75NA, pump (Stokes) power at the sample 6 mW (7.5 mW), 0.1 ms pixel dwell time, averaged to 3s per spectral point.

The results in Figure 2 show that mRhubarb720 exhibits the largest EPR-SRS effect, compared to emIRFP670, mIRFP670nano3, and mCherry, as expected from the resonant absorption wavelength being closer to the pump wavelength. Note that emIRFP670 and mIRFP670nano3 also use BV as chromophore but have an absorption peak λ_max_ of 644 and 645 nm, respectively (Figure 1c), hence blue-shifted compared to mRhubarb720 at 703 nm, with a frequency difference ν_e_-ν_p_ of about 3300 cm^–1^ compared to 2030 cm^–1^ for mRhubarb720. While similar bond types are present in mCherry’s chromophore, it has a different chromophore (Figure 1b) and a λ_max_ at about 590 nm (Figure 1c), so is further away from the pump wavelength in the SRL experiment, corresponding to a frequency difference of 4754 cm^–1^. As a result, the SRL peak signal of mRhubarb720 is about 10-fold higher than for mCherry (Figure 2c).

The EPR-SRL signal is expected to scale as the Raman cross-section. For a single dominant transition, the frequency dependent factor is given by^28^ *F*_*A*_ = (ν_*p*_ – ν_*v*_)^2^ (ν_*e*_^2^ + ν_*p*_^2^) (ν_*e*_^2^ – ν_*p*_^2^)^−2^ and is shown in Figure S4 for the experimental parameters used here. The Raman cross-section and thus the SRL signal scales with *F*_*A*_^2^, and compared to mRhubarb720 a 7-fold reduction for IRFP670 and a 29-fold reduction for mCherry is expected. These factors have the same trend but are somewhat larger than the experimentally measured ones. The frequency dependent factor was explored for a range of fluorophores and pump wavelengths in the literature,^16,26^ also showing a somewhat weaker dependence than expected, and variations between fluorophores. With increasing frequency, an increase of the coupling to the vibration expressed as vibronic energy (eq 4b in ref 28) might be expected, due to a reduced extension of the electronic excitation over the conjugated chains, counteracting the decreasing resonance enhancement and explaining the observed behavior.

Considering the excitation and detection conditions in our experiment, we have estimated the detection sensitivity limit for mRhubarb720 (see also Section S10). The SRL relative intensity modulation is found to be ΔI/I = 2.6 × 10^–5^ at 1 mM protein concentration. This is similar to the value reported for the ATTO740 dye previously,^17^ when scaled to the laser power and concentration conditions used here. The photon shot-noise limit in our experiment (as obtained from the measured DC current) is found to be 1.2 × 10^–8^ / $\sqrt{\mathrm{Hz}}$ . At 500 Hz bandwidth (corresponding to 1 ms per pixel) this equates to 1.9 × 10^–7^ (for one quadrature of the detection) which results in a detection limit of 7.2 μM concentration. The linear scaling of the signal with concentration was verified using a concentration series (see Figure S21), and the experimental noise was determined to be 8.0 μM, close to the estimated shot-noise limit. When further scaling to the powers used by Wei and Min^17^ we find a shot-noise limited detection equating to 452 nM. This is comparable to the detection limit for ATTO740 of 250 nM reported previously.^17^ Hence, mRhubarb720 appears to be an SRS tag with a signal strength comparable to the infrared dye ATTO740 characterized previously,^17^ while bringing the much sought after genetic encoding capability. We then used mRhubarb720 for cell imaging, to demonstrate its application as genetically encoded Raman tag.

We employed mRhubarb720 as a classical fusion tag to facilitate imaging in mammalian cells (Section S7). Specifically, mRhubarb720 was fused to the C-terminal of the histone protein H2B, a structural protein of eukaryotic chromosomes. In HeLa cells (Figure 3a), quantitative differential interference contrast (qDIC) microscopy (for imaging methods see Section S5) shows the cell structure with a “heart shaped” nucleus, nucleoli, and the cytosol containing small lipid droplets. SRL (Figure 3b-d), epi-fluorescence (Epifluor) microscopy (Figure 3e) and forward fluorescence (Fluor; Figure 3f-g) of the same cell were measured. Epi-fluorescence highlights the nucleus as expected due to the localization of H2B-mRhubarb720. SRL and forward fluorescence were measured simultaneously under pulsed laser excitation, using galvo beam scanning for imaging, and a photomultiplier with appropriate bandpass filters for fluorescence (see Section S5). SRL measurements used 1640 cm^–1^ as center IFD to optimally drive vibrational resonances. They were alternating between IFD wavenumbers of 1620 cm^–1^, the peak of the mRhubarb720 SRL, and 1660 cm^–1^ where there is weaker mRhubarb720 SRL (see Figure 2a blue lines), but significant SRL from endogenous cellular components, e.g., C=C bonds in lipids.^29^

**Figure 3:**
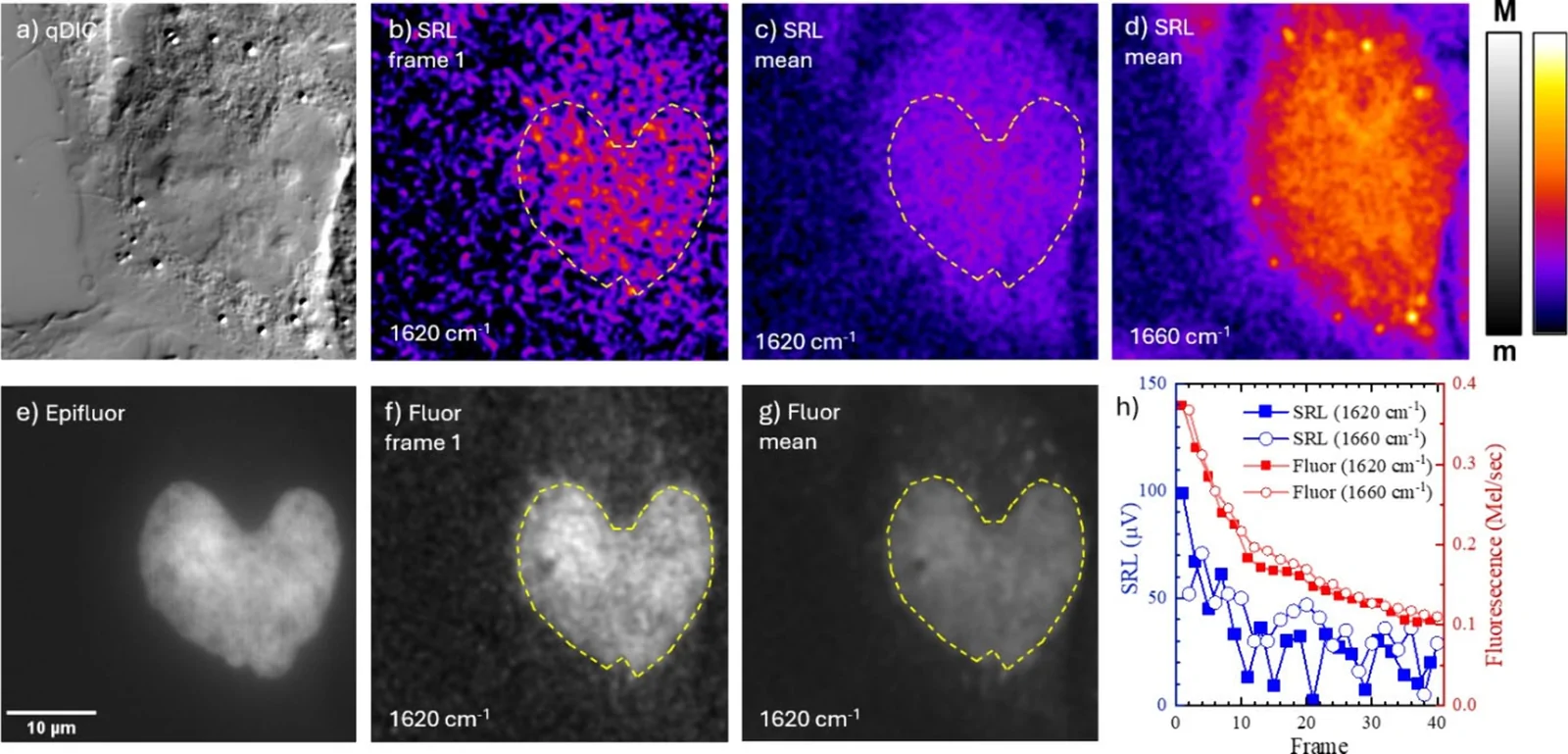
Imaging HeLa cell H2B-labeled mRhubarb720 expression. (a) qDIC, (b-d) SRL, (e) widefield epi-fluorescence (Epifluor) and (f-g) forward detected fluorescence (Fluor) are shown, as indicated. Gray and color scales are linear from m to M as follows: qDIC (m = 0.7rad to M = 0.7rad), SRL (m = 0.9 mV to M = 2.2 mV), Epifluor (m = 0pe to M = 4kpe, where pe are photoelectrons), Fluor (m = 0 Mpe/s to M = 1 Mpe/s). SRL and Fluor are taken over 40 frames, alternating between 1620 and 1660 cm^–1^, 0.01 ms pixel dwell time, 50 × 50 μm at 463 × 463 points, about 2s per frame. qDIC and Epifluor is taken before CRS imaging. The mean is taken over the 20 frames using the wavenumber given. Microscope objective 60× 1.27NA, pump (Stokes) power at the sample 7 mW (12 mW). Centre IFD 1640 cm^–1^. (h) The spatially averaged SRL and Fluor over the heart-shaped nucleus, as a function of frame number, separated for the two wavenumbers. The SRL has been background-subtracted using the nonfluorescent cell regions (see Figure S14).

Forward fluorescence detection for mRhubarb720 in solution (Figure S6) suggests that this fluorescence is dominated by one-photon absorption of the pump-only beam, which is possible due to the preresonant condition. As mentioned, albeit the pump beam has an energy below the absorption peak of mRhubarb720, at room temperature the thermal population of higher energy vibrational states enables one-photon absorption of the pump into the first electronic excited state, and subsequent fluorescence (see Section S8). Upon imaging, forward fluorescence accordingly shows the nucleus geometry, similar to wide-field epi-fluorescence. There is significant fluorescence bleaching during the time-course acquisition repeated over several frames (see red lines in Figure 3h), indicating that one and two-photon absorption of mRhubarb720 creates real excitations leading to photobleaching.

The first SRL frame acquired at 1620 cm^–1^ shows a more specific nucleus contrast, though with low signal-to-noise (Figure 3b). The mean SRL signal at 1620 cm^–1^ averaged over all acquired frames is weak and shows a cell outline where the nucleus cannot be clearly distinguished (Figure 3c). At 1660 cm^–1^ the cell outline is more visible, and lipid droplets appear (Figure 3d). The SRL signal at 1620 cm^–1^ exhibits a similar photobleaching behavior as fluorescence (see the blue filled squares in Figure 3h). For SRL acquired at 1660 cm^–1^ this bleaching is slightly reduced but still present (blue circles in Figure 3h). The observed SRL signal at 1620 cm^–1^ is about 0.08 mV in the first frame, corresponding to a concentration of mRhubarb720 of about 10 μM, estimated from the solution measurements (Figure 2). Similar results are shown in Figure S15 on another HeLa cell example.

Therefore, when fixed inside cells, mRhubarb720 photobleaching limits its application in fluorescence and in SRS imaging, as detailed in Section S9. Notably, while the demonstration of EPR-SRS in solution is promising (as shown in Figure 2), in this scenario proteins are free to diffuse in and out of the focal volume and refill any bleached product. For mRhubarb720 fixed in cells, we studied the dependence of photobleaching on imaging scan speeds (see Figures S18–S20), suggesting that cumulative effects, such as long-lived triplet states and local heating, are not the cause of the observed bleaching. The main bleaching mechanism is likely due to excited state absorption during a single pulse, initiated by one or two-photon absorption. Therefore, future efforts will have to address FP designs with reduced photobleaching and hence more robust upon EPR-CRS excitation conditions, for example by suppressing two-photon absorption, as discussed in Section S8.

In conclusion, we have demonstrated the first steps to genetically encoding probes for use in cellular based electronically preresonance coherent Raman imaging. The relatively narrow spectral line widths of these probes, coupled with the electronic preresonant effect, exhibiting signal enhancements comparable to those shown in the literature for synthetic ATTO dyes, open a promising avenue toward supermultiplexing CRS with genetic encoding. As the basis for the probes are existing fluorescent proteins, the transition of FPs to SRS imaging is relatively straightforward from a cell biology point of view. The next step is to further develop these probes as well as microscopy setups. Microscopy developments include SRS set-ups featuring pump wavelengths in the visible, so that FPs with chromophores absorbing in the blue to red range can be used, as well as SRS multiplex acquisition, for detection of multiple probes. Furthermore, SRS implementations using lower repetition rate lasers and faster beam scanning could enable reduction of cumulative photobleaching. Probe developments include reducing two-photon absorption cross sections, to suppress spectrally broad background and photobleaching, and further expanding the utility of such probes via the incorporation of electronically coupled Raman bonds vibrating in the biologically silent region, e.g. via reprogrammed genetic code approaches.^30–33^

## Supplementary Material



## Data Availability

Information on the data underpinning the results presented here, including how to access them, are shared via FigShare (https://figshare.com/s/3503c0259bd34acd54e3).

## Notes

Detailed methods, gene sequences, Sections S1−S10, Supporting Tables S1–S2 and Supporting Figures S1–S21 (PDF)

## Abbreviations

BV: biliverdin Xi-α
qDIC: quantitative Differential interference contrast
FP: fluorescent protein
EPR-CRS: electronically preresonant coherent Raman scattering
IRFP: infrared fluorescent protein
SRS: stimulated Raman scattering
SRL: stimulated Raman loss
ν_p_: pump laser frequency
ν_S_: Stokes laser frequency
ν_e_: chromophore electronic absorption peak frequency
