# Supplementary material for: Genetically Encoding Stimulated Raman-Scattering Probes for Cell Imaging Using Infrared Fluorescent Proteins
Source: J Phys Chem Lett. 2026 Aug 11;17(34):9862–8. doi: 10.1021/acs.jpclett.6c02085 (PMC13528288; doi:10.1021/acs.jpclett.6c02085)
Supplement: Supplementary file 1 [file jz6c02085_si_001.pdf]

Supporting Information for Publication

Genetically Encoding Stimulated Raman-Scattering Probes for Cell Imaging Using Infrared Fluorescent Proteins

David Regan[1], Ozan Aksakal[1], John J.K. McLarnon[1], Athena Zitti[1], Magdalena Lipka-Lloyd[2], Pierre J. Rizkallah[2], Anna J. Warren[3], Peter D. Watson[1], Wolfgang Langbein[4], D. Dafydd Jones[1], Paola Borri[1]

1. School of Biosciences, Cardiff University, Cardiff CF10 3AX, UK.
2. School of Medicine, Cardiff University, Cardiff CF14 4XN, UK.
3. Diamond Light Source Ltd, Harwell Science and Innovation Campus, Didcot OX11 0DE, UK.
4. School of Physics and Astronomy, Cardiff University, Cardiff CF24 3AA, UK.

Section S1 Gene sequences

emiRFP670

Near-IR FP
ATGGGGAGCCACCATCACCATCACCATGGCAGATCTATGGCGGAAGGCTCCGTCGCCAGGCAGCCTG
ACCTCTTGACCTGCGAACATGAAGAGATCCACCTCGCCGGCTCGATCCAGCCGCATGGCGCGCTTCTG
GTCGTCAGCGAACATGATCATCGCGTCATCCAGGCCAGCGCCAACGCCGCGGAATTTCTGAATCTCGG
AAGCGTACTCGGCGTTCCGCTCGCCGAGATCGACGGCGATCTGTTGATCAAGATCCTGCCGCATCTCG
ATCCCACCGCCGAAGGCATGCCGGTCGCGGTGCGCTGCCGGATCGGCAATCCCTCTACGGAGTACTGC
GGTCTGATGCATCGGCCTCCGGAAGGCGGGCTGATCATCGAACTCGAACGTGCCGGCCCGTCGATCG
ATCTGTCAGGCACGCTGGCGCCGGCGCTGGAGCGGATCCGCACGGCGGGTTCACTGCGCGCGCTGT
GCGATGACACCGTGCTGCTGTTTCAGCAGTGCACCGGCTACGACCGGGTGATGGTGTATCGTTTCGAT
GAGCAAGGCCACGGCCTGGTATTCTCCGAGTGCCATGTGCCTGGGCTCGAATCCTATTTCGGCAACCG
CTATCCGTCGTCGACTGTCCCGCAGATGGCGCGGCAGCTGTACGTGCGGCAGCGCGTCCGCGTGCTG
GTCGACGTCACCTATCAGCCGGTGCCGCTGGAGCCGCGGCTGTCGCCGCTGACCGGGCGCGATCTCG
ACATGTCGGGCTGCTTCCTGCGCTCGATGTCGCCGTGCCATCTGCAGTTCCTGAAGGACATGGGCGTG
CGCGCCACCCTGGCGGTGTCGCTGGTGGTCGGCGGCAAGCTGTGGGGCCTGGTTGTCTGTCACCATT
ATCTGCCGCGCTTCATCCGTTTCGAGCTGCGGGCGATCTGCAAACGGCTCGCCGAAAGGATCGCGAC
GCGGATCACCGCGCTTGAGAGCTAA

DNA (T7 promoter for HO-1)
GAGCTCGGCGCGCCTGCAGGTCGACAAGCTTGCGGCCGCATAATGCTTAAGTCGAACAGAAAGTAAT
CGTATTGTACACGGCCGCATAATCGAAATTAATACGACTCACTATAGGGGAATTGTGAGCGGATAACAA
TTCCCCATCTTAGTATATTAGTTAAGTATAAGAAGGAGATATACAT

HO-1
ATGACGAATCTTGCGCAGAAACTCAGGTATGGAACCCAACAGAGCCATACCTTGGCAGAGAACACCG
CATACATGAAGTGCTTCCTTAAAGGCATTGTTGAACGTGAACCTTTTAGACAGCTGCTCGCCAATTTGT
ACTACCTTTATTCCGCACTGGAGGCCGCACTCCGGCAGCATCGCGACAATGAGATCATCAGTGCAATCT
ATTTTCCTGAACTGAATCGTACGGACAAGCTCGCAGAGGATCTGACGTATTATTACGGTCCGAATTGGC
AGCAAATCATTCAGCCAACACCGTGCGCCAAAATATATGTAGATCGATTGAAAACGATTGCTGCATCGG

AGCCGGAATTGCTTATCGCTCACTGTTATACCCGCTATCTTGGTGATTTGTCGGGCGGCCAAAGTCTTA
AAAACATTATTCGTTCAGCACTGCAGTTGCCCGAAGGTGAAGGGACGGCTATGTATGAGTTCGACAGC
CTGCCAACACCGGGAGATCGCCGGCAGTTTAAAGAAATCTACAGGGATGTGCTTAATAGCCTGCCACT
GGACGAAGCAACTATTAATAGGATCGTAGAAGAAGCTAACTATGCTTTTTCACTGAACCGCGAAGTAAT
GCATGATCTGGAGGACCTGATCAAAGCAGCGATAGGCGAGCATACCTTTGATCTGCTTACCAGACAAG
ATCGCCCAGGTTCGACCGAAGCTCGATCGACGGCAGGTCACCCGATTACGCTTATGTTTGTGAATAACT
CGAGTCTGGTAAAGAAACCGCTGCTGCGAAATTTGAACGCCAGCACATGGACTCGTCTACTAGCGCAG
CTTAATTAA

miRFP670nano3

Near-IR FP
ATGGGGAGCCACCATCACCATCACCATGGCAGATCTGCAGCTGGTACCATGGCAAACCTGGACAAGAT
GCTGAACACCACCGTGACCGAGGTGCGCAAGTTCCTGCAAGCGGACAGAGTGTGCGTGTTCAAGTTC
GAGGAAGATTACTCCGGCACCGTCTCGCACGAAGCCGTGGACGACAGATGGATTAGCATCCTGAAGA
CCCAGGTGCAGGACAGATACTTCATGGAAACCAGAGGCGAGGAATACGTCCACGGCAGATACCAGGC
CATCGCCGACATCTACACAGCCAATCTGGTCGAGTGCTACAGAGACCTGCTGATCGAGTTTCAGGTGC
GGGCCATTCTGGCTGTCCCCATCCTGCAAGGCAAGAAGCTGTGGGGCCTGCTGGTGGCCCACCAACT
GGCCGGCCCTCGGGAGTGGCAGACCTGGGAAATCGACTTCCTGAAACAGCAAGCCGTGGTGATGGG
CATCGCCATCCAGCAGAGCTGA

DNA (T7 promoter for HO-1)
CTGAATTCGAAGCTTGGCTGTTTTGGTGGATGAGAGAAGATTTTCAGCCTGATACAGATTAAATCAGA
ACGCAGAAGCGGTCTGATAAAACAGAATTTGCCTGGCGGCAGTAGCGCGGTGGTCCCACCTGACCCC
ATGCCGAACTCAGAAGTGAAACGCCGTAGCGCCGATGGTAGTGTGGGGTCTCCCCATGCGAGAGTAG
GGAACTGCCAGGCATCAGTATACACTCCGCTATCGCTACGTGACTGGGTCATGGCTGCGCCCCGACAC
CCGCCAACACCCGCTGACGCGCCCTGACGGGCTTGTCTGCTCCCGGCATCCGCTTACAGACAAGCTGT
GACCGTCTCCGGGAGCTGCATGTGGAGCTCGGCGCGCCTGCAGGTCGACAAGCTTGCGGCCGCATAA
TGCTTAAGTCGAACAGAAAGTAATCGTATTGTACACGGCCGCATAATCGAAATTAATACGACTCACTATA
GGGGAATTGTGAGCGGATAACAATTCCCCATCTTAGTATATTAGTTAAGTATAAGAAGGAGATATACAT

HO-1
ATGACGAATCTTGCGCAGAAACTCAGGTATGGAACCCAACAGAGCCATACCTTGGCAGAGAACACCG
CATACATGAAGTGCTTCCTTAAAGGCATTGTTGAACGTGAACCTTTTAGACAGCTGCTCGCCAATTTGT
ACTACCTTTATTCCGCACTGGAGGCCGCACTCCGGCAGCATCGCGACAATGAGATCATCAGTGCAATCT
ATTTTCCTGAACTGAATCGTACGGACAAGCTCGCAGAGGATCTGACGTATTATTACGGTCCGAATTGGC
AGCAAATCATTCAGCCAACACCGTGCGCCAAAATATATGTAGATCGATTGAAAACGATTGCTGCATCGG
AGCCGGAATTGCTTATCGCTCACTGTTATACCCGCTATCTTGGTGATTTGTCGGGCGGCCAAAGTCTTA
AAAACATTATTCGTTCAGCACTGCAGTTGCCCGAAGGTGAAGGGACGGCTATGTATGAGTTCGACAGC
CTGCCAACACCGGGAGATCGCCGGCAGTTTAAAGAAATCTACAGGGATGTGCTTAATAGCCTGCCACT
GGACGAAGCAACTATTAATAGGATCGTAGAAGAAGCTAACTATGCTTTTTCACTGAACCGCGAAGTAAT
GCATGATCTGGAGGACCTGATCAAAGCAGCGATAGGCGAGCATACCTTTGATCTGCTTACCAGACAAG
ATCGCCCAGGTTCGACCGAAGCTCGATCGACGGCAGGTCACCCGATTACGCTTATGTTTGTGAATAACT
CGAGTCTGGTAAAGAAACCGCTGCTGCGAAATTTGAACGCCAGCACATGGACTCGTCTACTAGCGCAG
CTTAAC

The larger buffer in the DNA between the proteins was required by TwistBiosciences.

H2B-mRhubarb720

ATGCCAGAGCCAGCGAAGTCTGCTCCCGCCCCGAAAAAGGGCTCCAAGAAGGCGGTGACTAAGGC
GCAGAAGAAAGGCGGCAAGAAGCGCAAGCGCAGCCGCAAGGAGAGCTATTCCATCTATGTGTACA
AGGTTCTGAAGCAGGTCCACCCTGACACCGGCATTTCGTCCAAGGCCATGGGCATCATGAATTCGTT
TGTGAACGACATTTTCGAGCGCATCGCAGGTGAGGCTTCCCGCCTGGCGCATTACAACAAGCGCTCG
ACCATCACCTCCAGGGAGATCCAGACGGCCGTGCGCCTGCTGCTGCCTGGGGAGTTGGCCAAGCAC
GCCGTGTCCGAGGGTACTAAGGCCATCACCAAGTACACCAGCAGCTCTAAGGATCCACCGGTCGCC
ACCATGGGCAGCAGCAGCCAGGATCCGATGGCTGAAGGATCCGTCGCCAGGCAGCCTGACCTCTTG
ACCTGCGACGATGAGCCGATCCATATCCCCGGTGCCATCCAACCGCATGGACTGCTGCTCGCCCTCG
CCGCCGACATGACGATCGTTGCCGGCAGCGACAACCTTCCCGAACTCACCGGACTGGCGATCGGCG
CCCTGATCGGCCGCTCTGCGGCCGATGTCTTCGACTCGGAGACGCACAACCGTCTGACGATCGCCTT
GGCCGAGCCCGGGGCGGCCGTCGGAGCACCGATCACTGTCGGCTTCACGATGCGAAAGGACGCAG
GCTTCATCGGCTCCTGGCATCGCCATGATCAGCTCATCTTCCTCGAGCTCGAGCCTCCCCAGCGGGAC
GTCGCCGAGCCGCAGGCGTTCTTCCGCCACACCAACAGCGCCATCCGCCGCCTGCAGGCCGCCGAA
ACCTTGGAGAGCGCCTGCGCCGCCGCGGCGCAAGAGGTGCGGAAGATTACCGGCTTCGATCGGGT
GATGATCTATCGCTTCGCCTCCGACTTCAGCGGCGAAGTGATCGCAGAGGATCGGTGCGCCGAGGT
CGAGTCAAAACTAGGCCAGCACTATCCTGCCTCAGATATTCCGGCGCAGGCCCGTCGGCTCTATACC
ATCAACCCGGTACGGATCATTCCCGATATCAATTATCGGCCGGTGCCGGTCACCCCAGACCTCAATC
CGGTCACCGGGCGGCCGATTGATCTTAGCTTCGCCATCCTGCGCAGCGTCTCGCCCGTCCATCTGGA
ATTCATGCGCAACATAGGCATGCACGGCACGATGTCGATCTCGATTTTGCGCGGCGAGCGACTGTG
GGGATTGATCGTTTGCCATCACCGAACGCCGTACTACGTCGATCTCGATGGCCGCCAAGCCTGCGAG
CTAGTCGCCCAGGTTCTGGCCCGGGCGATCGGCGTGATGGAAGAGTAA

Lifeact-mRhubarb720

ATGGGCGTGGCCGACTTGATCAAGAAGTTCGAGTCCATCTCCAAGGAGGAGGGGGATCCACCGGTC
GCCACCATGGGCAGCAGCAGCCAGGATCCGATGGCTGAAGGATCCGTCGCCAGGCAGCCTGACCTC
TTGACCTGCGACGATGAGCCGATCCATATCCCCGGTGCCATCCAACCGCATGGACTGCTGCTCGCCC
TCGCCGCCGACATGACGATCGTTGCCGGCAGCGACAACCTTCCCGAACTCACCGGACTGGCGATCG
GCGCCCTGATCGGCCGCTCTGCGGCCGATGTCTTCGACTCGGAGACGCACAACCGTCTGACGATCGC
CTTGGCCGAGCCCGGGGCGGCCGTCGGAGCACCGATCACTGTCGGCTTCACGATGCGAAAGGACG
CAGGCTTCATCGGCTCCTGGCATCGCCATGATCAGCTCATCTTCCTCGAGCTCGAGCCTCCCCAGCGG
GACGTCGCCGAGCCGCAGGCGTTCTTCCGCCACACCAACAGCGCCATCCGCCGCCTGCAGGCCGCC
GAAACCTTGGAGAGCGCCTGCGCCGCCGCGGCGCAAGAGGTGCGGAAGATTACCGGCTTCGATCG
GGTGATGATCTATCGCTTCGCCTCCGACTTCAGCGGCGAAGTGATCGCAGAGGATCGGTGCGCCGA
GGTCGAGTCAAAACTAGGCCAGCACTATCCTGCCTCAGATATTCCGGCGCAGGCCCGTCGGCTCTAT
ACCATCAACCCGGTACGGATCATTCCCGATATCAATTATCGGCCGGTGCCGGTCACCCCAGACCTCA
ATCCGGTCACCGGGCGGCCGATTGATCTTAGCTTCGCCATCCTGCGCAGCGTCTCGCCCGTCCATCT
GGAATTCATGCGCAACATAGGCATGCACGGCACGATGTCGATCTCGATTTTGCGCGGCGAGCGACT
GTGGGGATTGATCGTTTGCCATCACCGAACGCCGTACTACGTCGATCTCGATGGCCGCCAAGCCTGC
GAGCTAGTCGCCCAGGTTCTGGCCCGGGCGATCGGCGTGATGGAAGAGTAA

Section S2 Protein production and purification

Plasmids containing the near-IR FPs (pET-DUET-1-mRhubarb720-HO [Addgene: #141201]; custom pET28a+ vectors for emiRFP670, miRFP670nano3 with HO1 (TwistBiosciences) (see Section S1 for gene sequences for each construct) were used. Near-IR constructs were transformed into chemically competent *E.coli* BL21(DE3) (New England Biolabs). For mCherry, a pBAD expression system was used [Addgene: #54630]; transformed into chemically competent *E.coli* TOP10 (New England Biolabs). All constructs were plated on LB agar ampicillin (50 μg/ml) and incubated at 37°C overnight. Selected colonies were then picked and incubated in 5 mL 2xYT ampicillin (50 μg/ml) cultures overnight at 37°C. The 5 ml overnight cultures were then used to inoculate 0.5 L 2xYT ampicillin (50 μg/ml) cultures. For the IRFPs, cultures were incubated at 37°C until an OD_{600} of 0.4-0.8 was reached, upon which 0.5 mM IPTG was added to induce IRFP expression. The cultures were then incubated for a further 24 h at 22°C with shaking. For mCherry, cultures were incubated at 37°C until an OD_{600} of 0.4-0.8 was reached, upon which 1% (w/v) L-Arabinose was added to induce mCherry expression. These cultures were then incubated for a further 18 h at 25°C with shaking.
Harvested cells were resuspended in 50 mM Tris buffer (pH 8.0) and lysed using a French press; cell lysate was clarified by centrifugation and supernatant was collected. The FPs were purified using a His-trap HP nickel affinity column (GE Healthcare) in conjunction with ATKA Purifier. The His-trap column was equilibrated with 50 mM Tris (pH 8.0), 300 mM NaCl and 10 mM Imidazole and eluted with 50 mM Tris (pH 8.0), 300 mM NaCl with 500 mM Imidazole. Pooled FPs samples were then subjected to size exclusion chromatography using a HiLoad 26/600 Superdex 200 column, where the column was equilibrated with 50mM Tris (pH 8.0). Elution was monitored as specific wavelengths, depending on the λ_{max} of the FP. Protein purity was validated using SDS-PAGE analysis.

Section S3 Protein electronic excitation characterisation

Each purified sample was subject to a DC Protein Assay (BioRAD) to determine protein concentrations using the manufacturer's guidelines. BSA (0.2-1.5 mg/ml) was used as the concentration standard. UV-Vis spectroscopy was performed using a Cary 60 spectrophotometer (Agilent Technologies). Characterisations used 10 μM of each sample for absorbance measurements across 200–800 nm. Extinction coefficients were calculated using the Beer-Lambert law.

Table S1. Spectral properties of IRFPs used in this study.

IRFP	Determined here		Previously reported [a]	
	λ_{max} (nm)	ε ($mM^{-1}cm^{-1}$)	λ_{max} (nm)	ε ($mM^{-1}cm^{-1}$)
mRhubarb720	703	72.5	701[b]	95.0[b]
emIRFP670	644	23.2	642[c]	87.4[c]
miRFP670nano3	645	31.9	645[d]	129.0[d]

a, values reported in FPbase (https://www.fpbase.org/) [1]
b, Reported by Rogers et al. [2]
c, Reported by Matlashov et al. [3]
d, Reported by Oliinyk et al. [4]

Section S4 Crystallography

Crystallisation screens were set up using hanging-drop vapor diffusion method for the purified mRhubarbWT at 10 mg/mL, with 0.1 M MMT buffer (pH 5.0) and 25% (w/v) polyethylene glycol 1500. Micro-crystals of mRhubarb grew which were suitable for the microfocus macromolecular crystallography beamline VMXm[5] at Diamond Light Source. Crystals were prepared on cryo-electron microscopy grids, using a Leice EM GP2 plunge freezer, with vitrification in liquid ethane, before mounting on the VMXm beamline. 30° wedges of data were collected from multiple crystals, with an oscillation of 0.1° and exposure time of 0.2 s per image, with 100% transmission of beam. The data were collected at an energy of 21.326 keV (wavelength of 0.5814 Å). Each individual dataset was processed with DIALS[6], with the resulting processed data being input into xia2.multiplex[7] to merge and produce a complete dataset. Initial structure determination was carried out by Alphafold[8] with manual molecular replacement for the Biliverdin XI-alpha cofactor using CCP4[9] and COOT.[10] The refined structure of mRhubarbWT was visualised using PyMOL. Data collection and refinement statistics can be found in Table S2.

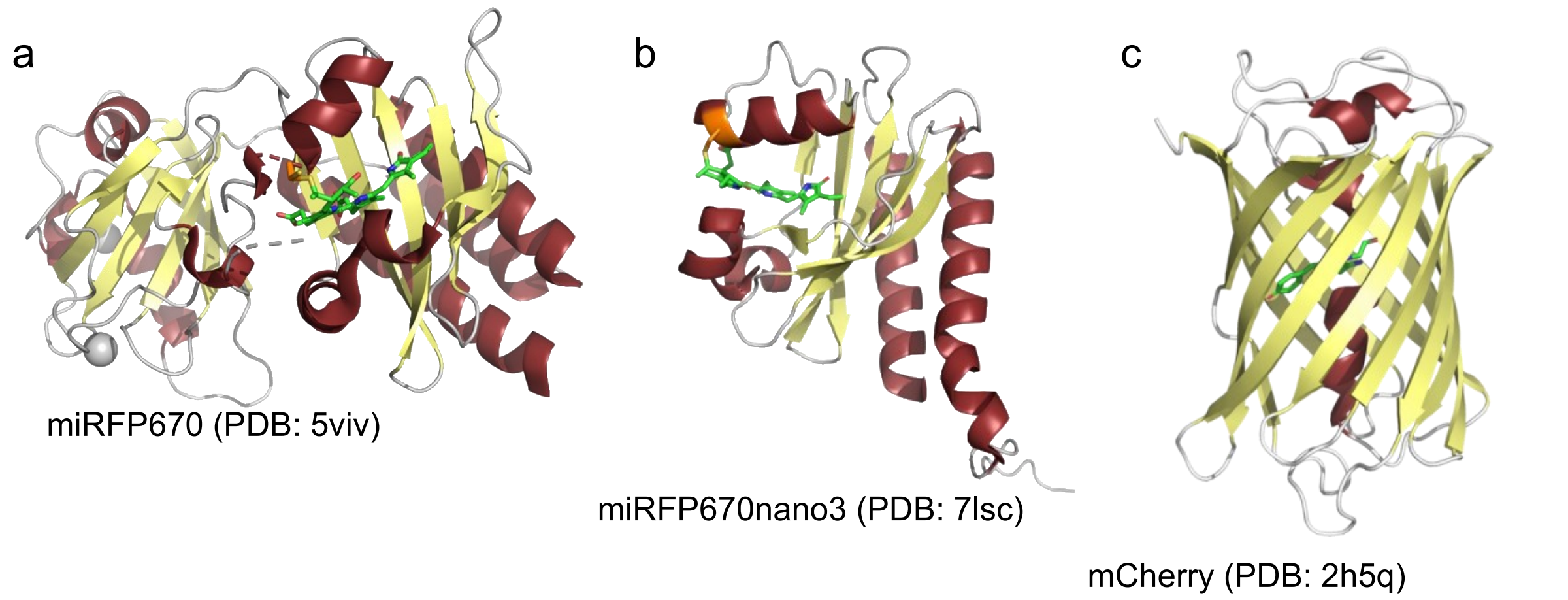

Figure S1. Structures of various infrared and red fluorescent proteins. (a) miRFP670 (PDB 5viv)[11], an engineered truncated IFRP miRFP670nano3 (PDB 7lsc)[4] and the β-barrel-type fluorescent protein with the autocatalytically forming chromophore, mCherry (PDB 2h5q)[12].

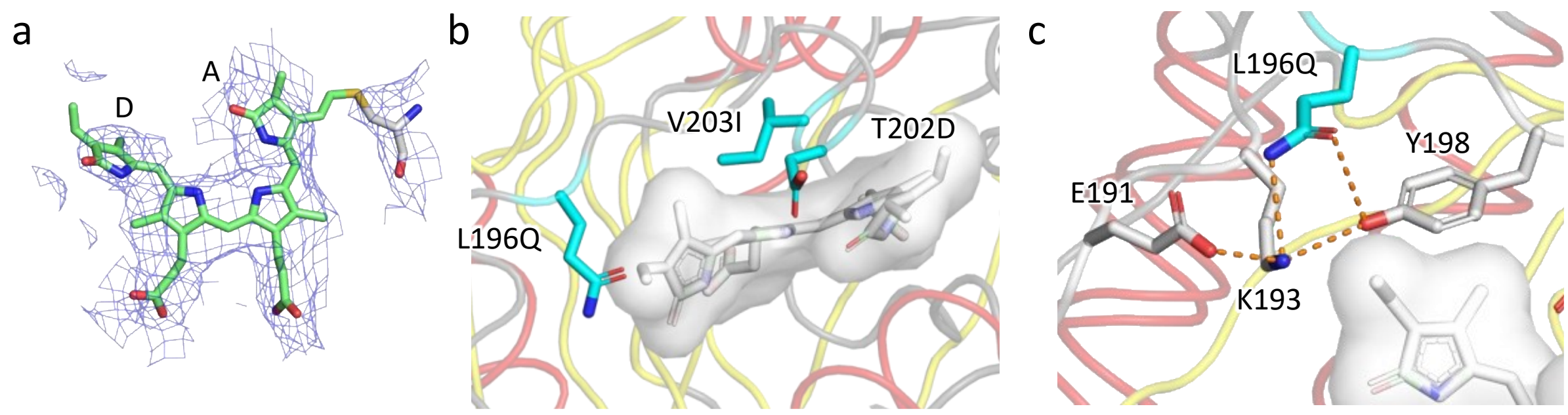

Figure S2. mRhubarb720 structural details. (a) the $2F_0$-F_c map of biliverdin set to 1.5 σ. The thioether link between ring A and C15 is shown for reference. (b) The positioning of key mutations generating mRhubarb720. (c) Potential polar interaction network between L196Q and local residues. We note the lack of electron density around the supposed thioether bond.

This could indicate that the bond is flexible and prone to movement, and/or the presence of unbound BV in the crystals measured.

Table S2. Data collection and refinement statistics for mRhubarb
* Three crystals were merged to produce this data set.
[1] Coordinate Estimated Standard Uncertainty in (Å), calculated based on maximum likelihood statistics.

PDB Entry	9F03
**Data Collection***	
Diamond Beamline	VMXm
Date	21 Jan 2022
Wavelength (Å)	0.58136
**Crystal Data (figures in brackets refer to outer resolution shell)**	
a,b,c (Å)	58.85, 58.85, 185.88
a,b,g (°)	90.0, 90.0, 90.0
Space group	$P\ 4_1\ 2_1\ 2$
Resolution (Å)	3.30 – 27.41
Outer shell	3.30 – 3.57
R-merge (%)	33.4 (164.5)
R-pim (%)	20.2 (106.7)
R-meas (%)	37.2 (179.3)
CC1/2	0.971 (0.315)
I / σ(I)	4.8 (0.7)
Completeness (%)	95.1 (90.7)
Multiplicity	4.2 (2.9)
Total Measurements	21,465 (2,786)
Unique Reflections	5,072 (953)
Wilson B-factor($Å^2$)	77.6
**Refinement Statistics**	
Refined atoms	2,382
Protein atoms	2,339
Non-protein atoms	43
Water molecules	0
R-work reflections	4,779
R-free reflections	261
R-work/R-free (%)	22.5 / 30.9
**rms deviations (ML target in brackets)**	
Bond lengths (Å)	0.008 (0.013)
Bond Angles (°)	1.448 (1.648)
[1]Coordinate error	0.892
Mean B value ($Å^2$)	86.0
**Ramachandran Statistics (PDB Validation)**	
Favoured/allowed/Outliers	230 / 46 / 28
%	75.7 / 15.1 / 9.2

Section S5 SRS, DIC, and fluorescence microscopy

Measurements were acquired using a home-built coherent Raman microscope setup described in our previous work[13] with simultaneous SRS, forward and epi confocal-fluorescence detection. A sketch of the set-up is shown in Fig. S3. SRS is measured in the form of stimulated Raman loss (SRL) at a pump wavelength of 820 nm. For SRS, the Stokes beam is amplitude modulated at 2.5 MHz using an acoustic-optic modulator (AOM). Pump and Stokes are recombined using a dichroic beam splitter (DBS1, Melles Griot LWP-45-RP808-TP1064-PW-1025-C) and raster-scanned on the sample using a galvo scanner and a 4f optical relay from the scanner to the microscope objective (MO), mounted onto a commercial inverted microscope stand (Nikon Ti-U). The sample is mounted on an xyz piezoelectric stage (MadCityLabs NanoLP200). The signal is collected by a 1.34 NA oil-immersion condenser (Nikon MEL41410) in transmission geometry. SRL is detected as an intensity modulation $\Delta I/I$ by a photodiode (PD, Hamamatsu S6976) using a short-pass filter F2 (Semrock FF01-945/SP and Thorlabs DMLP900R in reflection) to separate the pump from the Stokes beam, a home-built resonant preamplifier at 2.5 MHz, and a lock-in amplifier (Zurich Instruments HF2LI). To be able to use IFD centres below 1740 cm^{-1}, the short-pass filter F2 was later-on replaced with a stack of two Thorlabs FESH0850, to block the Stokes beam at wavelengths closer to the pump.

A dichroic beamsplitter (DBS2, Semrock FF776-Di01) in the detection was used to transmit/reflect signal above/below 776 nm, to separate SRL from fluorescence. The fluorescence signal was spectrally selected by using a bandpass filter F1 (Semrock FF01-675/67) transmitting 642-707 nm and detected by a photomultiplier (PMT- Hamamatsu H10770A-40). Note that this forward PMT detection is also sensitive to coherent anti-Stokes Raman scattering (CARS) in the wavenumber range above 1750 cm^{-1}, as observed for measurements carried out on the Tris buffer (Fig. S6). Epi-fluorescence in the range 560-750 nm is reflected by the dichroic beamsplitter DBS3 (Eksma) and filtered by F3 (Semrock FF02-650/100) transmitting 600-700 nm, focussed through an adjustable confocal pinhole PH, and detected by a photomultiplier E-PMT (Hamamatsu H10770-40). The PH was adjusted to a diameter of twice the Airy disk of the objective (equivalent to $2\times1.22\times650$ nm/NA at the sample). For confocal epifluorescence imaging, sample scanning instead of beam scanning is used.

Spectral focussing is implemented using glass blocks as described in our previous work.[13] Here, several 11 cm long H-ZF88 glass blocks were added to the setup to increase the maximum group delay dispersion and achieve a spectral resolution down to 6.7 cm^{-1}. The longer pulses used to achieve higher spectral resolution also reduce the signal, as is seen in Fig. S5, where SRS on a polystyrene (PS) calibration sample and mRhubarb720 is shown for 6.7, 11, and 21 cm^{-1} resolution. Improving the spectral resolution from 21cm^{-1} to 11 cm^{-1} results in better resolved mRhubarb720 resonances. Conversely, further decreasing to 6.7 cm^{-1} mostly reduces the signal, indicating that the resonance linewidth is larger than 6.7 cm^{-1}. Therefore, we used 11 cm^{-1} resolution for the measurements shown in this work. This resolution was obtained by adding 160 mm of H-ZF52A and 220 mm H-ZF88 in the pump beam, stretching the pulses from 106 fs to 3.1 ps intensity full-width at half-maximum (FWHM), and 160 mm of H-ZF52A and 330 mm H-ZF88 in the Stokes beam stretching the pulse from 129 fs to 2.7 ps.

Initially, the centre IFD was set to 1740 cm^{-1} using a Stokes wavelength of 956 nm. After replacing the filter F2, the centre IFD was set to 1640 cm^{-1} using a Stokes wavelength of

948 nm. Pump-Stokes two-photon absorption (TPA) manifests as a pump-loss in the SRL detection, and is clearly visible as a pump-Stokes temporal cross-correlation pulse intensity profile, since changing wavenumber equates to tuning the pump-Stokes relative time delay. This is shown in Fig. S10, plotting the SRL spectrum for mRhubarb720 measured at different centre IFDs. After changing the filter F2, lower centre IFD could be reached, and Fig. S8 shows the corresponding SRL spectra of mRhubarb720. The signal increased by about an order of magnitude using 1640 cm^{-1} with a Stokes wavelength of 948 nm, due to the more efficient resonant driving at pump-Stokes overlap.

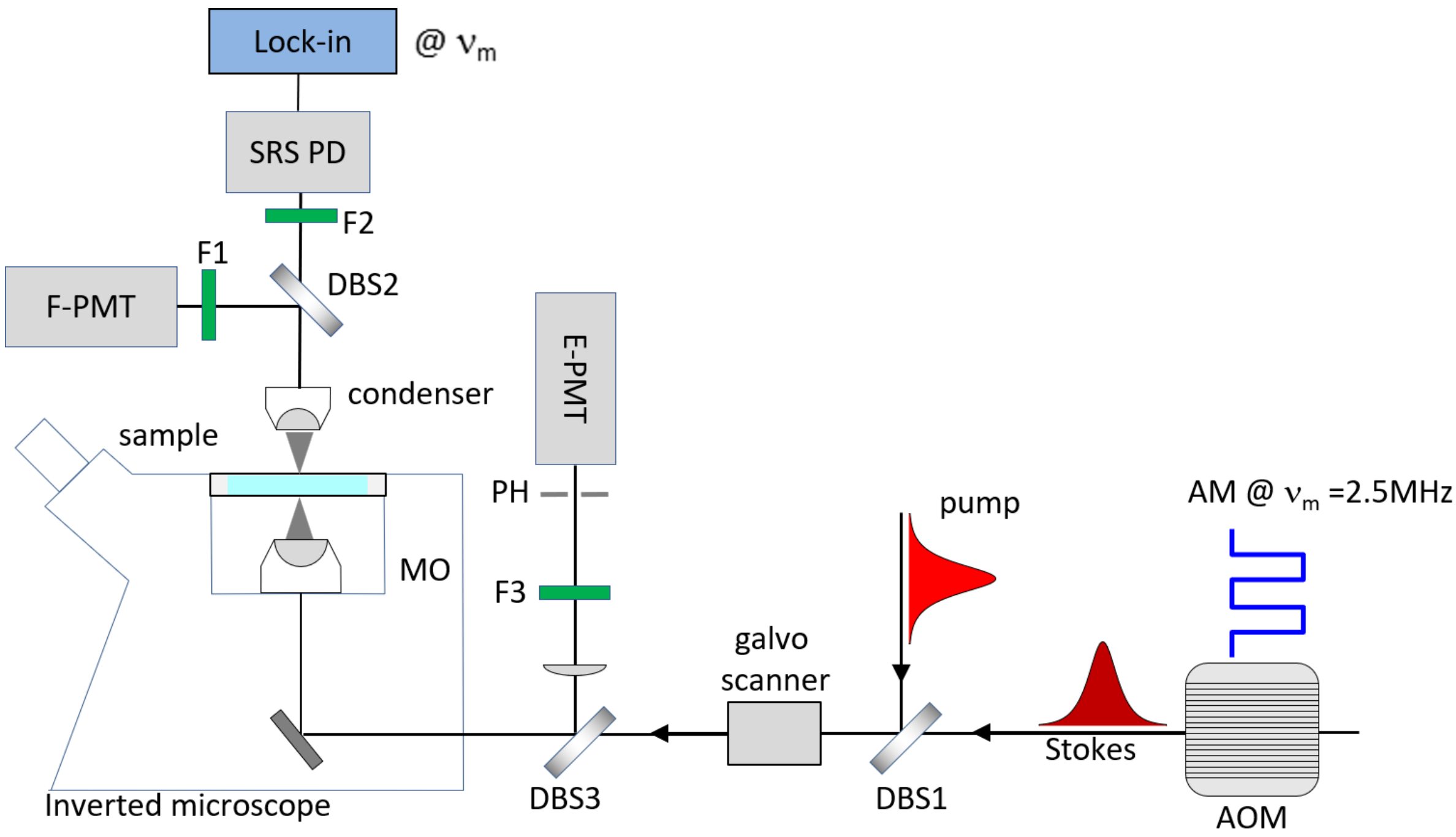

Figure S3. SRS microscopy set-up. The Stokes beam is amplitude modulated (AM) at 2.5 MHz using an acoustic-optic modulator (AOM). Pump and Stokes are recombined using the dichroic beam splitter DBS1 and raster scanned onto the sample using a galvo scanner. They are focused using a microscope objective (MO) mounted onto a commercial inverted microscope stand and the signal is collected by a 1.34 NA oil-immersion condenser in transmission geometry. SRL is detected as an intensity modulation by a photodiode (PD) using a short-pass filter F2 to separate the pump from the Stokes beam, a home-built resonant circuit at 2.5 MHz, and a lock-in amplifier. A dichroic beamsplitter DBS2 in detection is used to transmit/reflect signal above/below 776 nm, to separate SRL from fluorescence. The fluorescence signal is spectrally selected by using a bandpass filter F1 and detected by a photomultiplier F-PMT. Epifluorescence in the range 560-750 nm is reflected by the dichroic beamsplitter DBS3 filtered by F3, focussed through the adjustable confocal pinhole PH, and detected by a photomultiplier E-PMT.

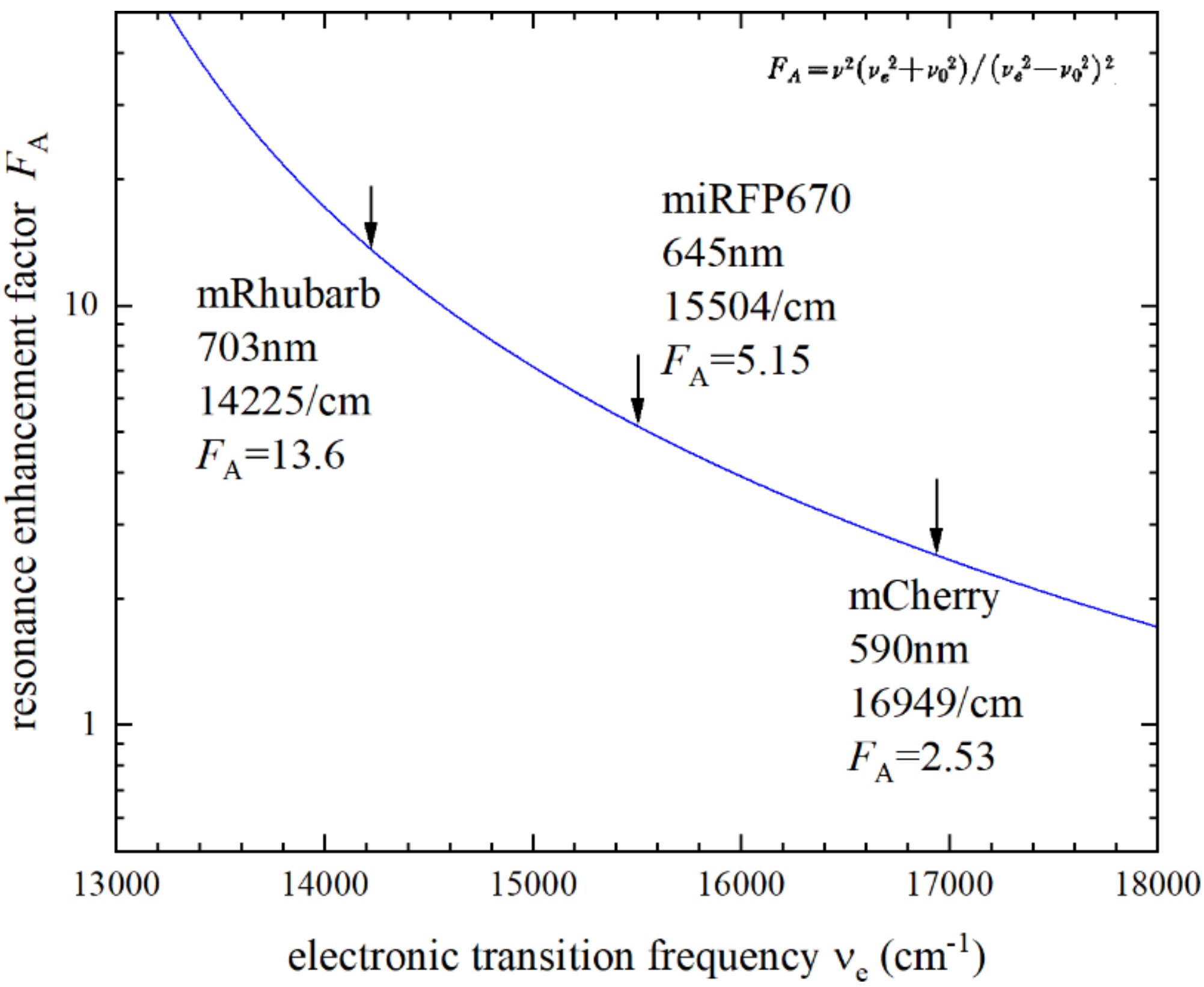

Figure S4. Resonance enhancement factor $F_A = \left(\nu_p - \nu_v\right)^2 \left(\nu_e^2 + \nu_p^2\right)\left(\nu_e^2 - \nu_p^2\right)^{-2}$ (see eq. 5 in Ref.15) for a pump wavelength of 820 nm and vibrational frequency of ν_v=1650 cm^{-1}**.** The electronic transition frequencies ν_e for the fluorophores measured are shown.

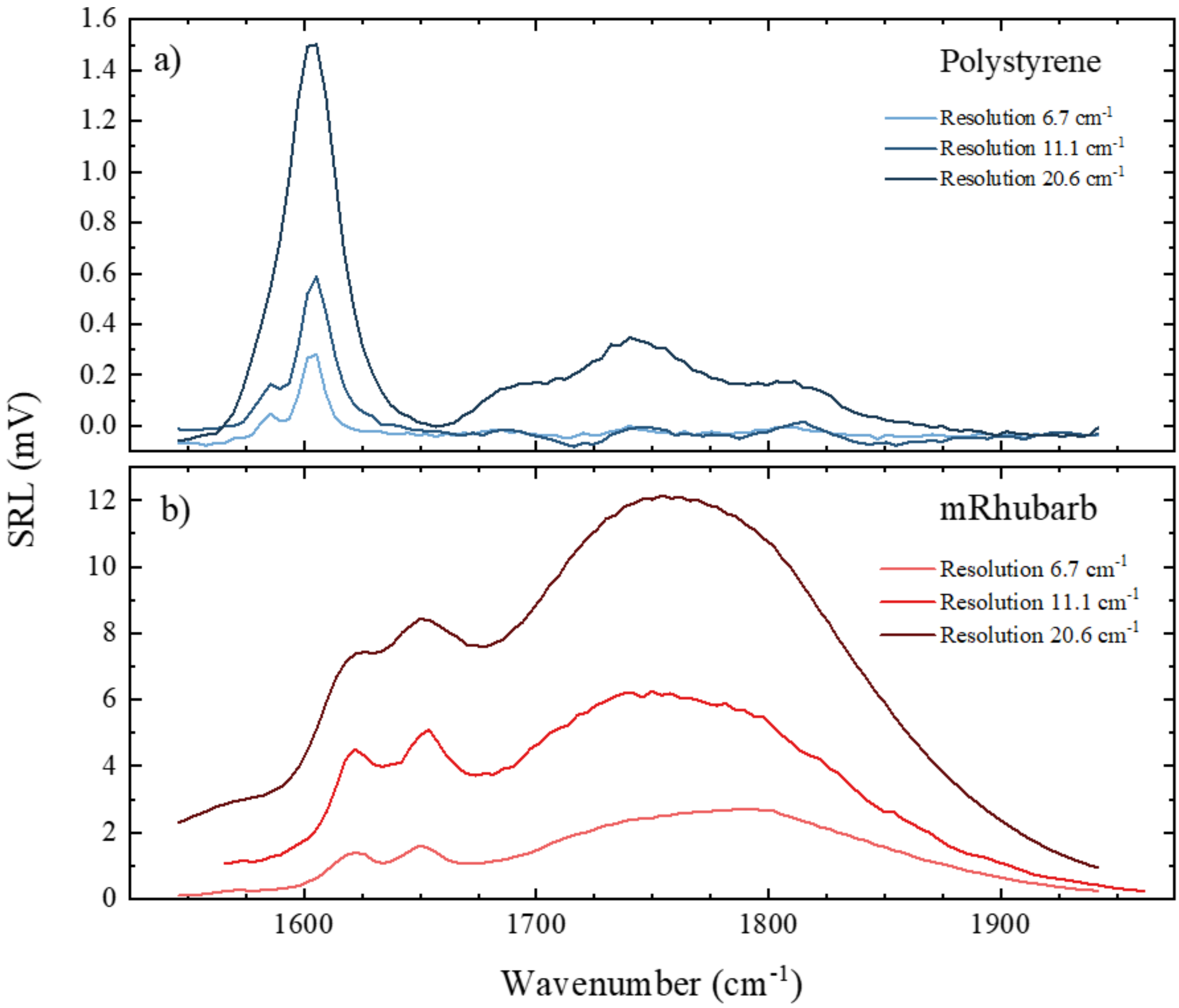

Figure S5. Comparison of the SRS spectra of a) polystyrene and b) mRhubarb720 in solution (1 mM), measured at different spectral resolutions, with 6 mW pump and 7.5 mW Stokes power at the sample, using the 20× 0.75 NA objective, and centre IFD 1740 cm^{-1}.

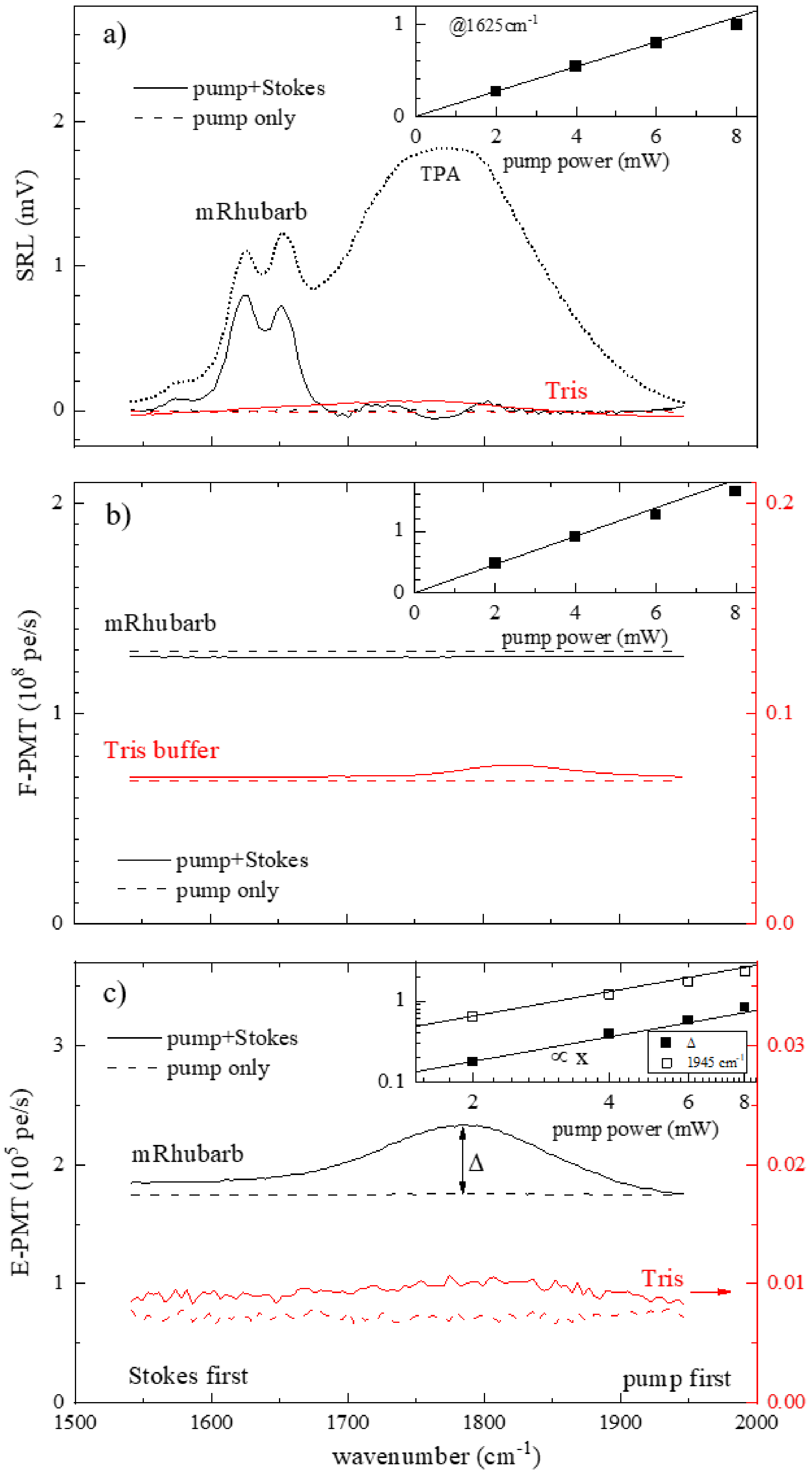

a)
pump+Stokes
pump only
@1625 cm-1
pump power (mW)
TPA
mRhubarb
Tris
SRL (mV)
b)
mRhubarb
pump power (mW)
Tris buffer
pump+Stokes
pump only
F-PMT (10^8 pe/s)
c)
pump+Stokes
pump only
Δ
1945 cm-1
∝ x
pump power (mW)
mRhubarb
Δ
Tris
Stokes first
pump first
E-PMT (10^5 pe/s)
wavenumber (cm-1)

Figure S6. Comparison between SRL (a), forward PMT (b) and confocal epi-PMT (c) signals for mRhubarb720 in solution (1 mM) at 6 mW pump and 7.5 mW Stokes power at the sample, 20× 0.75 NA objective. Insets show the dependence on pump-power of (a) vibrationally resonant response, (b) pump + Stokes, (c) Stokes. Experimental conditions are the same as in Fig. 2. The results without the Stokes beam (dashed), and for the Tris buffer (red) are also shown.

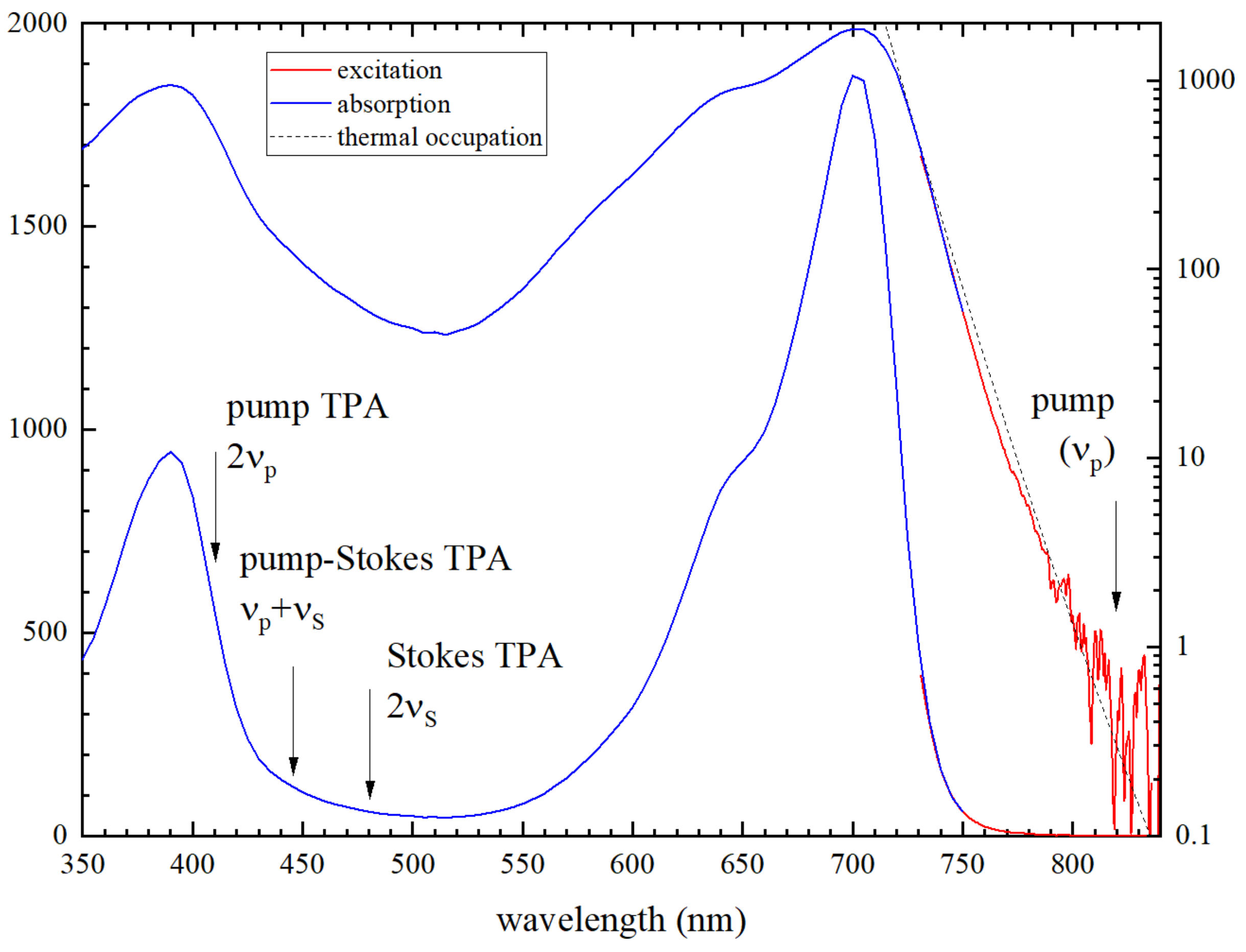

Figure S7. Absorption (blue) and fluorescence excitation spectra (red) of mRhubarb, on a linear and logarithmic scale, using arbitrary units. The absorption and excitation spectra have been scaled relatively, to match around 750 nm, with the excitation spectra allowing to show the absorption profile tail with 4 orders of magnitude dynamic range. A thermal occupation curve proportional to $\exp\left(h\nu/(k_B T)\right)$ with Planck's constant h, Boltzmann's constant k_B and the absolute temperature $T = 293\,\mathrm{K}$ is plotted as dashed line, showing good agreement with the absorption tail. The wavelength position of TPA from pump, Stokes and combined pump-Stokes photons are indicated.

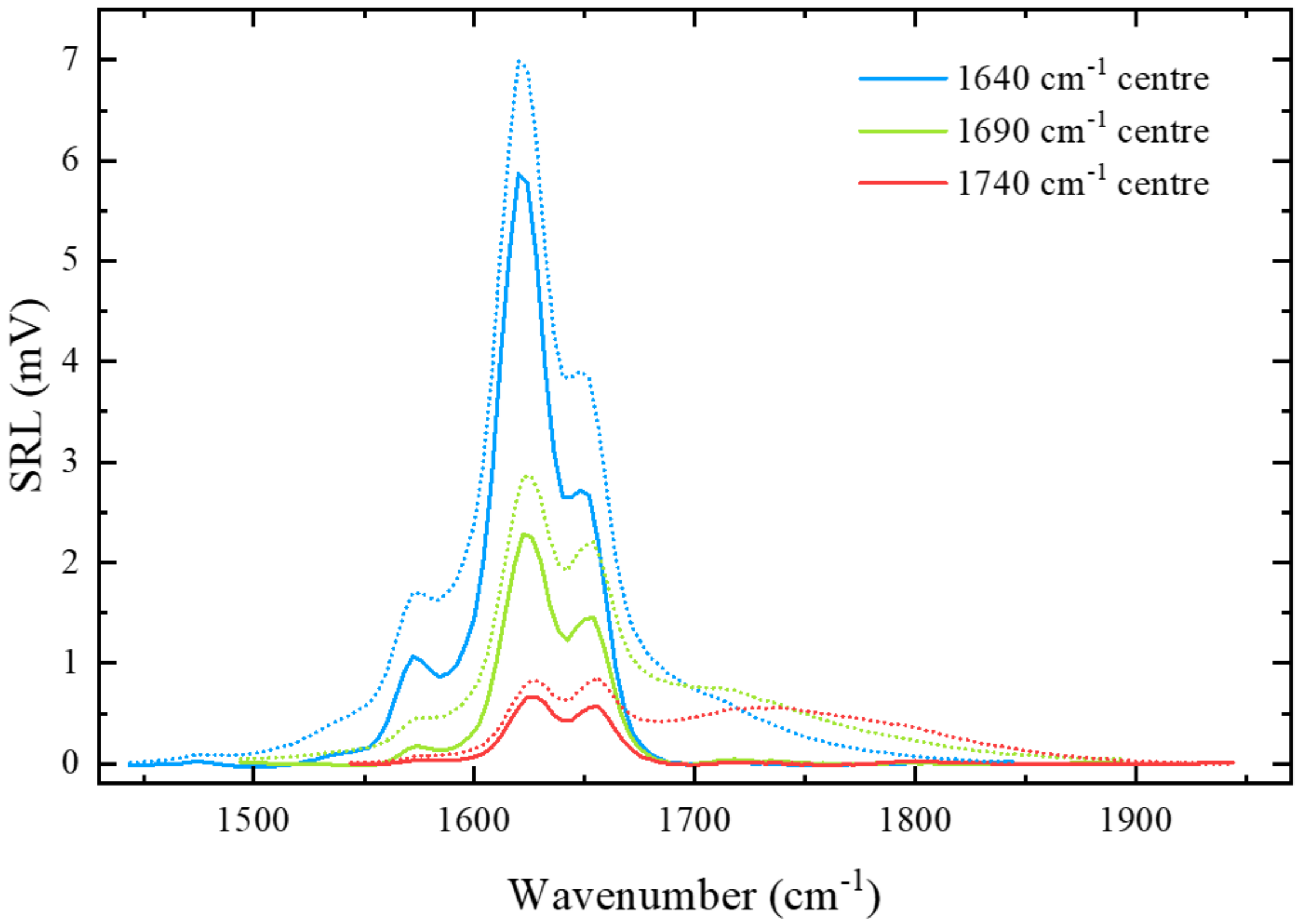

Figure S8. SRL signal of mRhubarb720 in solution (1 mM, with 6 mW pump and 7.5 mW Stokes power at the sample, 20× 0.75 NA objective, 11 cm^{-1} resolution) measured at different centre IFDs, as indicated, by using Stokes centre wavelengths of 956, 952 and 948 nm. Dotted lines as measured, solid lines TPA background subtracted.

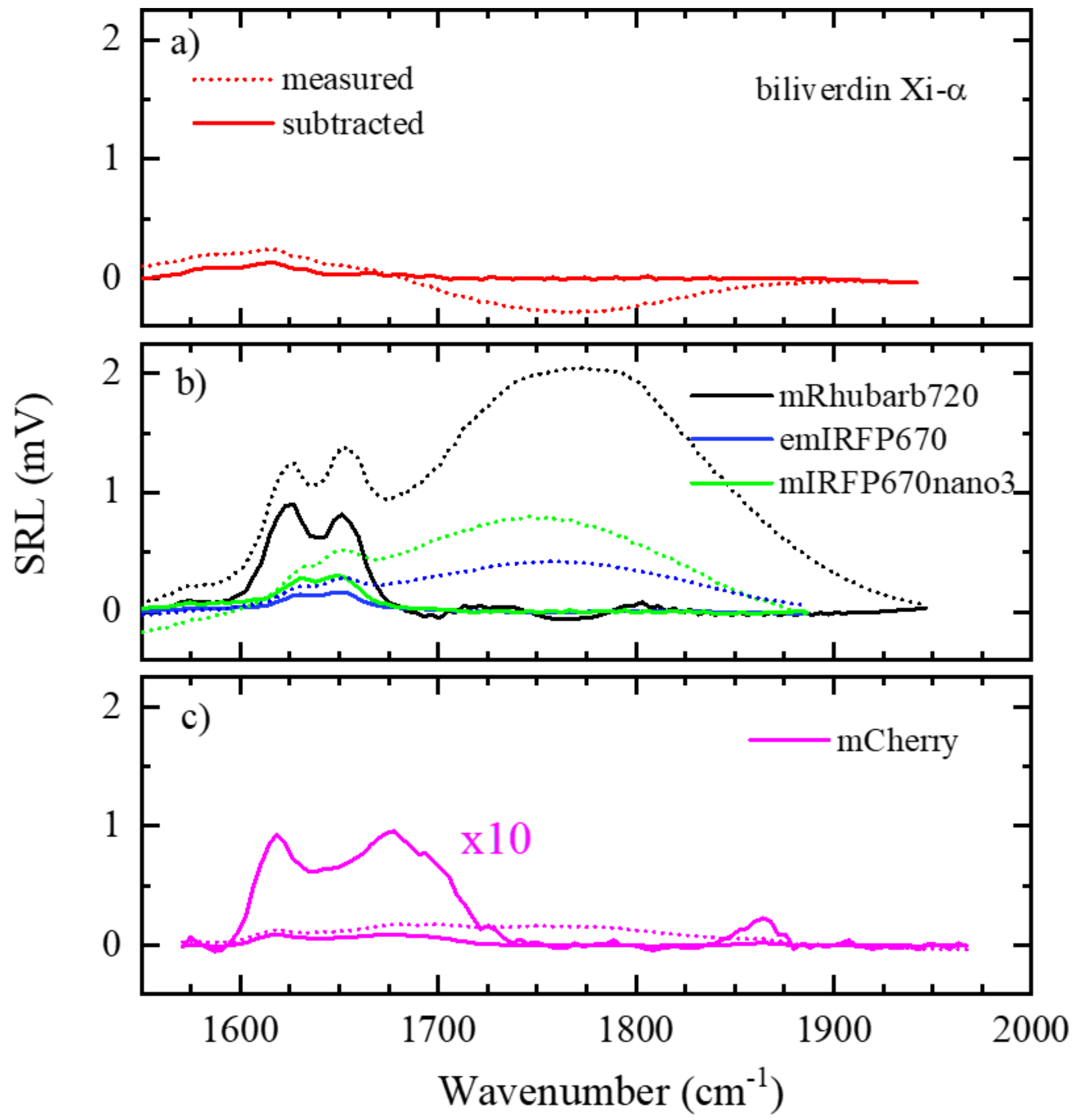

Figure S9. IRFPs SRL spectra before (dotted) and after (solid) subtraction of the pump-Stokes TPA effect. Same experimental conditions as in Fig. 2.

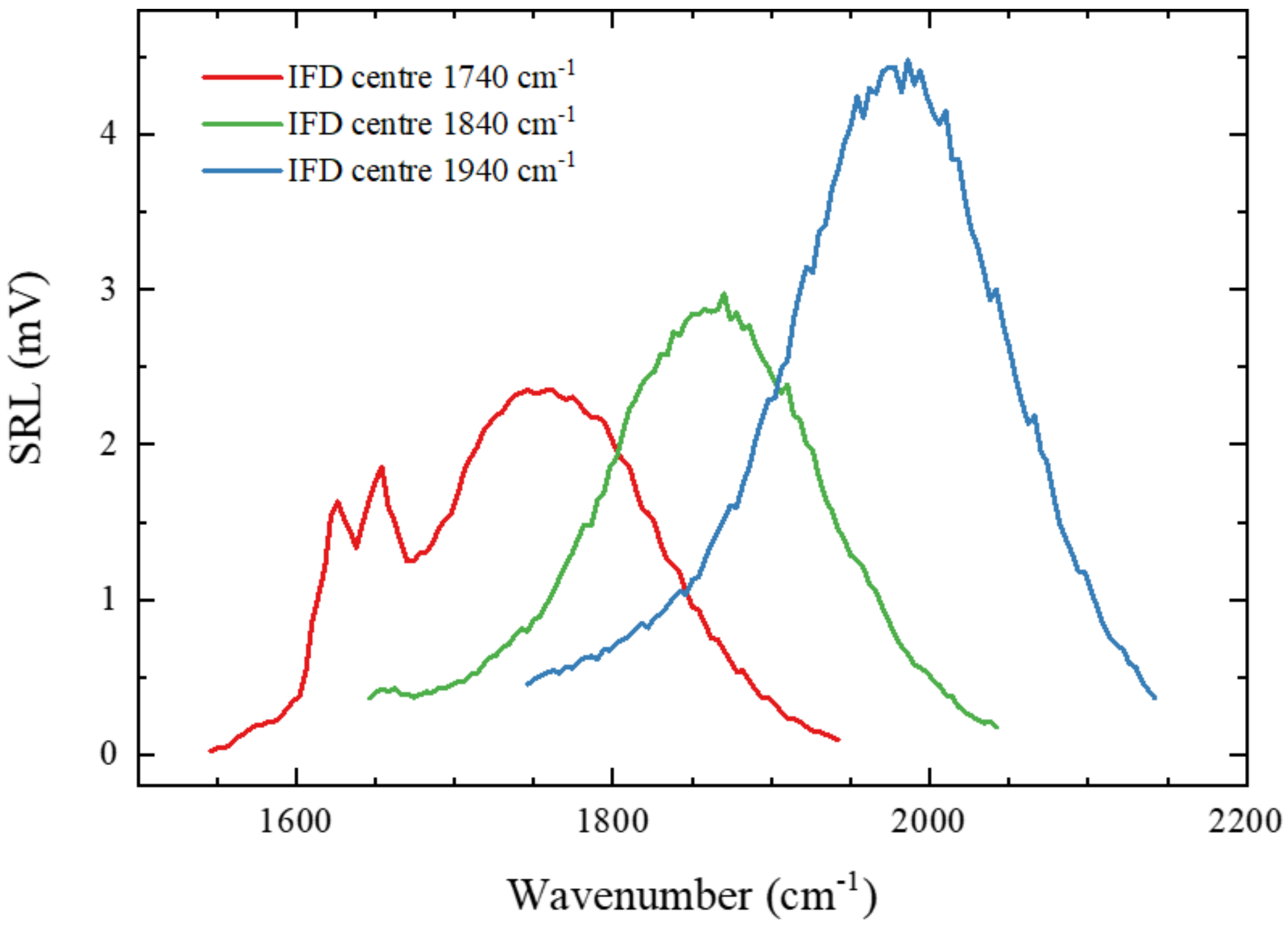

Figure S10. SRL signal of mRhubarb720 in solution (1 mM, with 6 mW pump and 7.5 mW Stokes power at the sample, 20× 0.75 NA objective, 11 cm^{-1} resolution) measured at different centre IFDs as indicated using Stokes centre wavelengths of 956, 966 and 975 nm. TPA results in an apparent SRL signal that follows the pump-Stokes temporal overlap (different wavenumbers correspond to different pump-Stokes relative delay times in spectral focussing).

To measure SRL spectra of the IRFPs in solution, we added 1 mM in 50 mM Tris buffer pH 8 into Grace BioLabs CultureWell™ gaskets having wells of 3 mm diameter and 1 mm depth, mounted between a slide and coverslip. A 20× 0.75 NA microscope objective (Nikon MRD00205) and 1.5 × tube lens was used, with the focus close to the centre of the well. SRL was measured for pump-Stokes delay scans over an IFD range of 1540 – 1940 cm^{-1} using 101 samples and 4 cm^{-1} step size. At each IFD, a spatial line scan over 10 µm with 1500 samples and 0.1 ms pixel dwell time was taken. The scans were then averaged over twenty repetitions and over the spatial dimension (corresponding to 3 s aggregated acquisition time per spectral point) to produce a plot of SRL signal against wavenumber. Pump (Stokes) power at the sample was 6 mW (7.5 mW) unless otherwise noted, with the Stokes power referring to 50% duty cycle modulated Stokes. An overview of the measured SRL for IRFPs in solution is shown in Fig. 2 and Fig. S9.

Simultaneous acquisition of SRL, forward and epi-detected fluorescence is shown for mRhubarb720 in Fig. S6. The forward-detected fluorescence (F-PMT, Fig. S6b) is strong, around 140×10^6 photoelectron/s (140 Mph/s), dominated by the emission due to the absorption of the pump-only beam, and scales linearly with the pump-power. It is therefore due to residual one-photon absorption owing to the pre-resonant condition as discussed in detail in section S8. While the pump beam has a frequency well below the absorption peak, at room temperature there is enough thermal population of vibrational states to enable one-photon absorption of the pump into the first electronic excited state, and subsequent fluorescence. The fluorescence detected by the F-PMT is dominated by the contribution from bulk volume in solution, since it is about 3 orders of magnitude higher than the confocal fluorescence detected by the E-PMT shown in Fig. S6c. The latter shows a pump-Stokes two-photon absorption effect which follows the pulse temporal cross-correlation profile while changing the delay time between pump and Stokes pulses in spectral focussing. This TPA-induced fluorescence (see Δ in Fig. S6c) is superimposed onto a pump-only contribution which scales linearly with the pump power, and thus is again dominated by a one-photon absorption as mentioned above. The lack of a significant pump-only TPA-induced fluorescence is due to its lower power and possibly a lower TPA cross-section for the pump as compared to the pump-Stokes case. Note that we do not see stimulated Raman excited fluorescence (SREF), since this should exhibit vibrational resonances versus wavenumbers[16] which are not observed in the detected fluorescence.

Control measurements without mRhubarb720 (Tris buffer only) show a weak SRL (see Fig. S6a), from spectrally broad vibrational resonances of water. The forward fluorescence contribution (see Fig. S6b) is also very small, about 20 times lower than with mRhubarb720. In this detection channel, there is a small contribution (around 1 Mpe/s) of non-resonant CARS, which is peaked around 1820 cm^{-1}, shifted compared to the centre IFD of 1740 cm^{-1} due to the filter cut-off supressing CARS detection for IFDs below 1800 cm^{-1}. The epi-detected confocal fluorescence (see Fig. S6c) is very weak (around 1 kpe/s), as there is no CARS in epi-direction for a homogeneous liquid.

As mentioned, the TPA of combined pump - Stokes results in a broad positive background SRL signal. To remove this background, the TPA profile is fitted using least squares to the data in the Curve Fitting Toolbox of MATLAB R2021a. We use a hyperbolic secant pulse shape typical for Kerr-lens mode locked laser pulses, superimposed to a sloped background, as given by

$$y = a\,\mathrm{sech}^2\left(\frac{x-b}{c}\right) + d + ex\,,$$

where *a*, *b*, and *c* are the amplitude, centre, and width of the pulse, and *d* and *e* are offset and slope of the background. Spectral regions showing strong Raman peaks were excluded from the fit, to isolate the TPA. For biliverdin, a weaker background with a bipolar shape was observed, likely due to a dominating Kerr lensing effect, and fitted using the function

$$y = a \tanh\left(\frac{x-b}{c}\right) + d + ex + f \tanh\left(\frac{x-g}{h}\right).$$

We note that we detect both the in-phase and in-quadrature components of the modulated SRL signal. In-quadrature components are signatures of long-lived responses on the timescale of the modulation period (400 ns), which can be of photothermal origin. We find a negligible signal, about two orders of magnitude below the in-phase signal, close to the noise level, showing that the signals observed here are not significantly affected by photothermal effects.

SRL and quantitative differential interference contrast (qDIC) images in cells were taken using a 60× 1.27 NA water immersion objective (Nikon MRD70650) and the 1× tube lens of the Ti-U inverted microscope stand. SRL was sequentially acquired as individual images at 1620 cm^{-1} and 1660 cm^{-1}, and this sequence was repeated 20 times. Pixel dwell times were 0.01 ms. Excitation powers at the sample were typically around 7 mW for pump and 12 mW (modulated) for Stokes.

The microscope was also equipped with wide-field epi-fluorescence excitation and detection. As excitation, we used a red LED centred at 634 nm having 21 nm width, delivered via a 3 mm diameter lightguide (Bluebox niji 7). In detection, we used a filter cube with an excitation filter (Edmund Optics 67-036) transmitting 603 to 678 nm, a 685 nm dichroic (Edmund Optics 67-085) and an emitter filter (Edmund Optics 87-758) transmitting 698 to 766 nm. The camera was an sCMOS (PCO.edge 5.5 RS) with 2560 x 2160 pixels. Data were taken using the maximum 2 s integration time, slow scan readout having a read noise of 1.6 e, and a gain of 0.5754 e/count. The camera was mounted at the eyepiece port of the Ti-U microscope.

qDIC images were acquired with the same camera, averaged over 128 acquisitions in fast scan readout, using a 100 W halogen lamp with a Thorlabs FB500-40 filter for transillumination. A de-Sénarmont compensator (T-P2 DIC Polariser HT MEN51941, Nikon), was used to control the phase offset, and a N2 DIC module (MEH52500, Nikon) was added in the condenser (HNAO MEL41410, Nikon) of 1.34 numerical aperture (NA), combined with a N2 DIC slider (MBH76190, Nikon) and a Ti-A-E DIC analyser block (MEN 51980, Nikon). The quantitative phase difference images were acquired and analysed as detailed previously[17,18] at a polariser angle of ±45°.

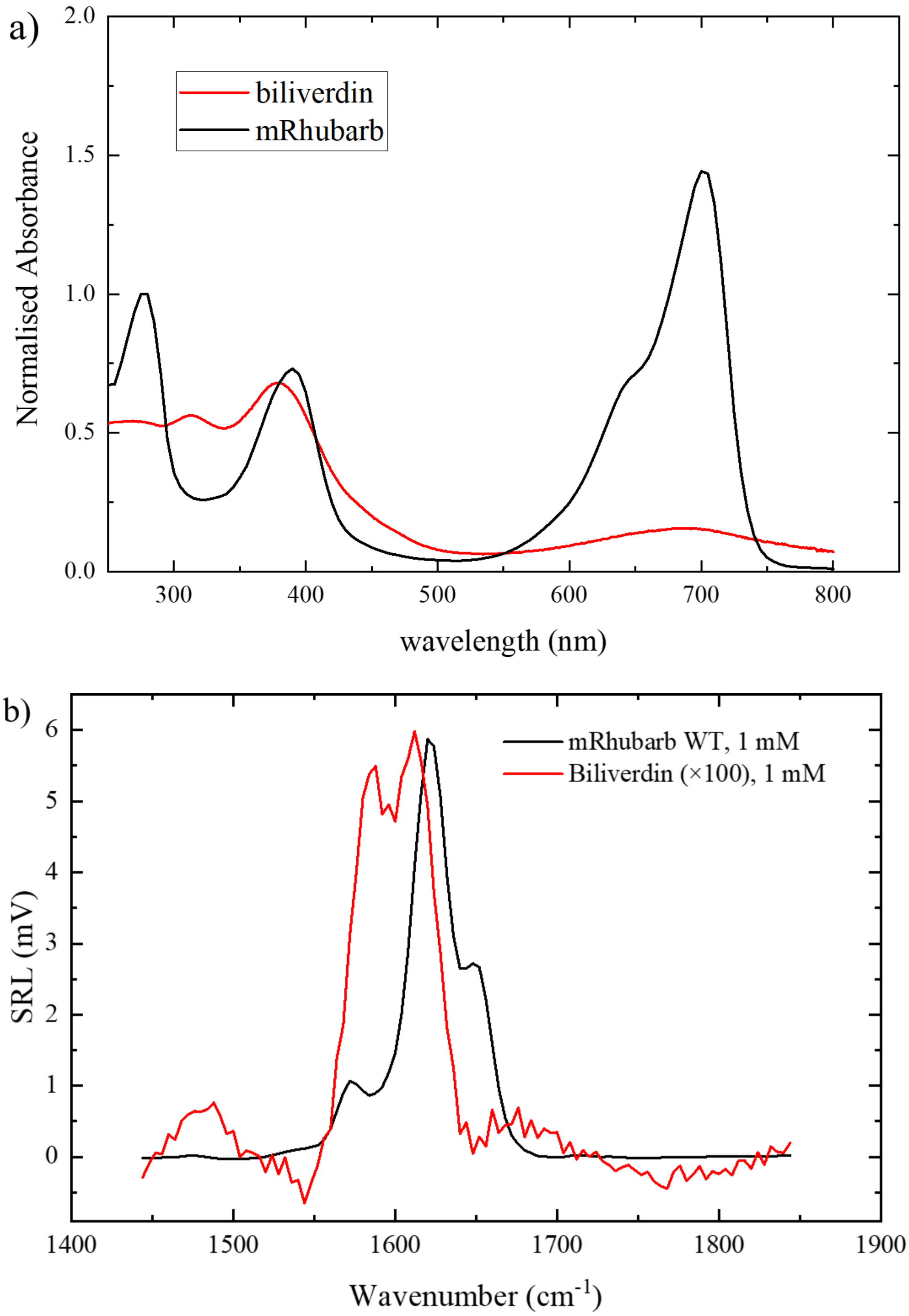

Figure S11. Comparison between free BV in 50 mM Tris and 50 mM NaOH (red) and BV bound to the protein component in mRhubarb720 in 50 mM Tris (black). a) Absorption spectrum b) SRL of 1mM free BV scaled by a factor of 100, and 1mM mRhubarb720. Microscope objective 20× 0.75 NA, pump (Stokes) power at the sample 6 mW (7.5 mW), 0.1 ms pixel dwell time, averaged to 3 s per spectral point, 1640cm^{-1} centre IFD.

Section S6 Density functional theory (DFT) and time dependent density function theory (TD DFT) analysis

Density functional theory (DFT) and time-dependent density function theory (TD-DFT) were performed using Gaussian09[19] to predict the Raman spectral characteristics of mRhubarb720 and BV. For the BV-bound model, BV and the R-groups of three key residues (R249, H285 and C15) were retained, while the remainder of the protein was removed in GaussView5[20] (Fig. S12). These key residues were held fixed during geometry optimisation to preserve key interactions with BV. Additional interacting residues were omitted to reduce computational cost.

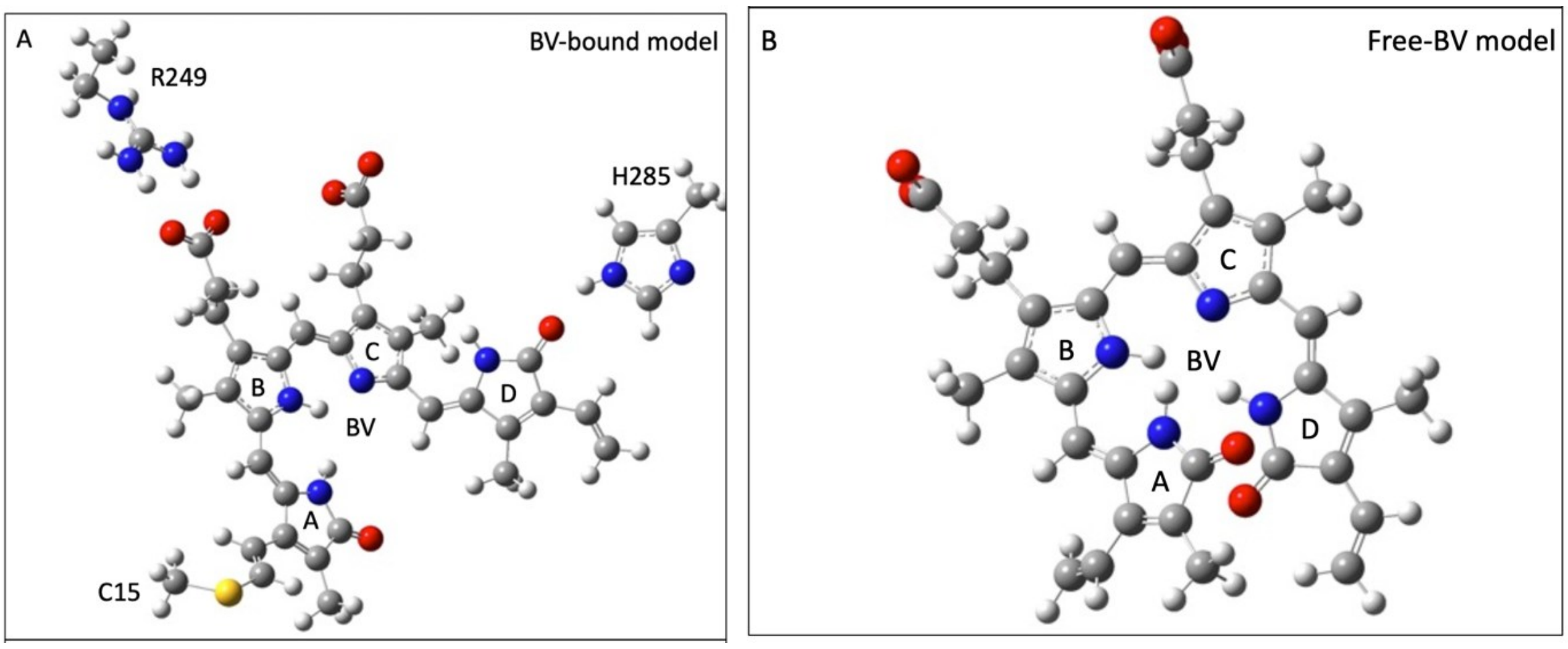

Figure S12. Geometry optimised models for TD DFT. (A) BV-bound model includes three interacting residues that are fixed in position whereas (B) free-BV model had no constraints. Both models are labelled with their ring nomenclature.

Initial coordinates for the BV-bound QM model were obtained from an MD-derived mRhubarb720 structure built from the mRhubarb720 crystal structure (PDB: 9F03). Atomic connectivity from this starting structure was preserved in Gaussian09 using geom=connectivity. Due to the resolution of the mRhubarb720 structure and the associated uncertainty in the protonation state of BV when protein-bound, BV was modelled with a deprotonated C-ring, consistent with the BV IX-α protonation state formed during heme catabolism[21]. The total charge/multiplicity used were −1/1 for BV-bound and −2/1 for Free-BV. The Polarisable Continuum Model (PCM: scrf=(solvent=water) was used to simulate the aqueous environment around the chromophore and interacting residues. The solvent, water, was modelled with a static dielectric constant (ϵ) of 78.3553. Gaussian09 constructed the solvent cavity using a scaled van der Waals cavity (scaling factor = 1.100) via the GEPOL algorithm with Universal Force Field (UFF) atomic radii. Polarisation charges were calculated via matrix inversion, using spherical Gaussians and a Karplus/York smoothing algorithm to ensure continuity across the molecular surface.[22–24] The keyword int=acc2e=11 was used to tighten the numerical accuracy of two-electron integral evaluation, reducing integral screening and improving DFT/TD-DFT numerical stability (particularly for a charged conjugated system with diffuse functions and PCM), at modest additional computational cost. For geometry optimisation, a three-step approach was used: HF/3-21G, B3LYP/6-31G and B3LYP/6-31G+(d,p). This strategy was chosen to balance computational efficiency with the accuracy of the resulting optimised geometries.

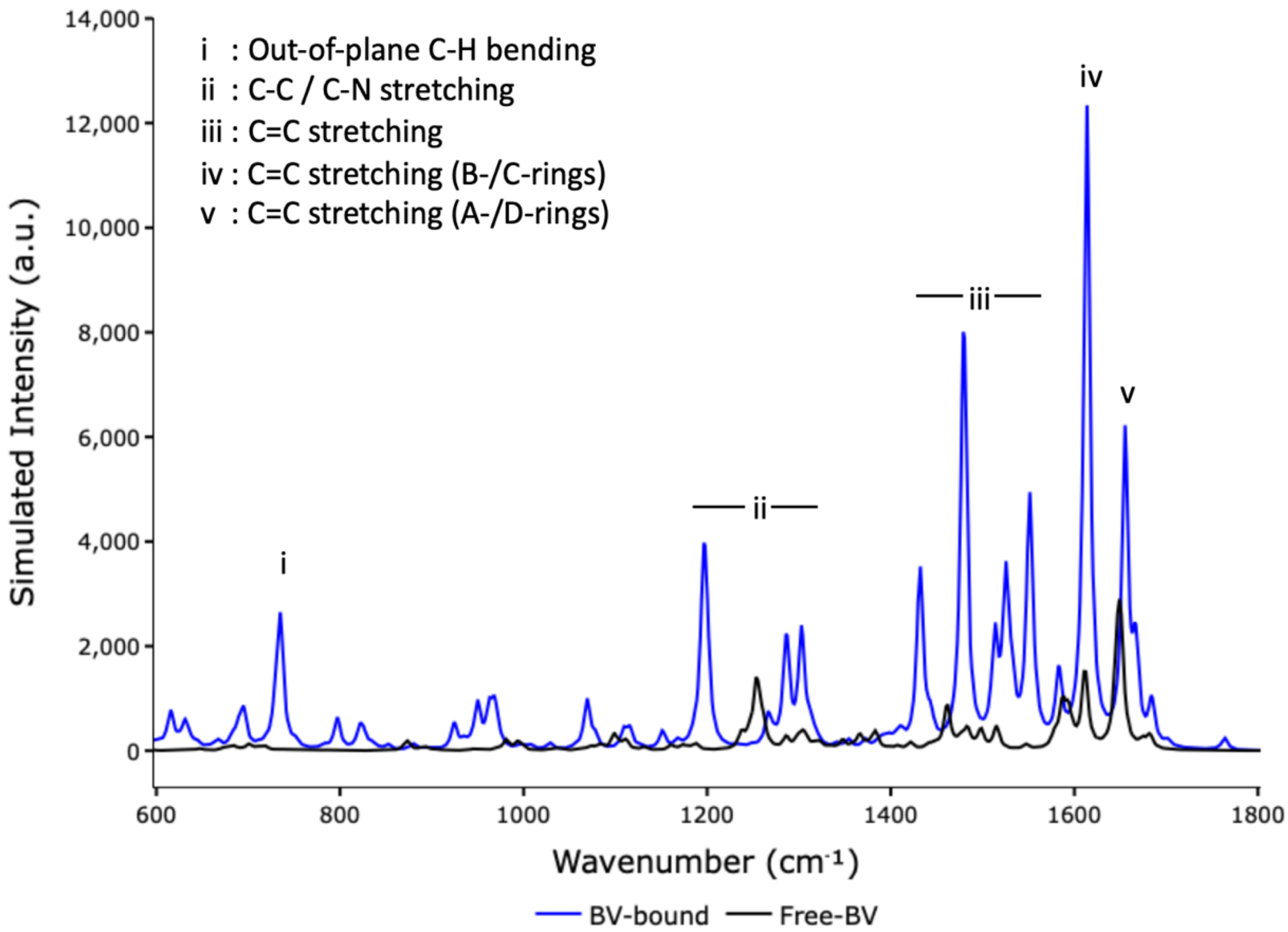

Figure S13. Fingerprint region of predicted Raman spectra. The simulated spectra of BV-bound (blue) is compared to free-BV (black). Selected peaks are labelled (i) Out-of-plane C-H bending, (ii) C-C/C-N stretching, (iii) C=C stretching, (iv) C=C stretching (B-/C-rings), (v) C=C stretching A-/D-rings). A scaling factor of 0.97 was applied to the wavenumbers for the simulated spectra[25].

TD-DFT excited-state calculations were performed with td=(nstates=10) at the B3LYP/6-31+G(d,p) level of theory (PCM water). Predicted Raman spectra were generated from freq=raman outputs using Gaussian broadening (GaussView default $\sigma = 10\ cm^{-1}$); We kept the Gaussian09 default incident wavelength of 1064 nm for Raman intensity calculations.

Section S7 Mammalian cell preparation

For mammalian cell imaging, HeLa cells (ATCC CCL-2) were grown on 25mm #1.5 borosilicate glass coverslips placed in 6 well tissue culture plates. Transfection was achieved using Fugene6 (Promega) according to manufacturer's instructions and allowed to express for 24hours. Coverslips were then washed in PBS before being fixed with 4% paraformaldehyde in PBS for 20 minutes. Following fixation, coverslips were washed in PBS and mounted on a cut down microscope slide using a 0.12 mm imaging gasket (Grace Biolabs) with PBS buffer in the resulting chamber.

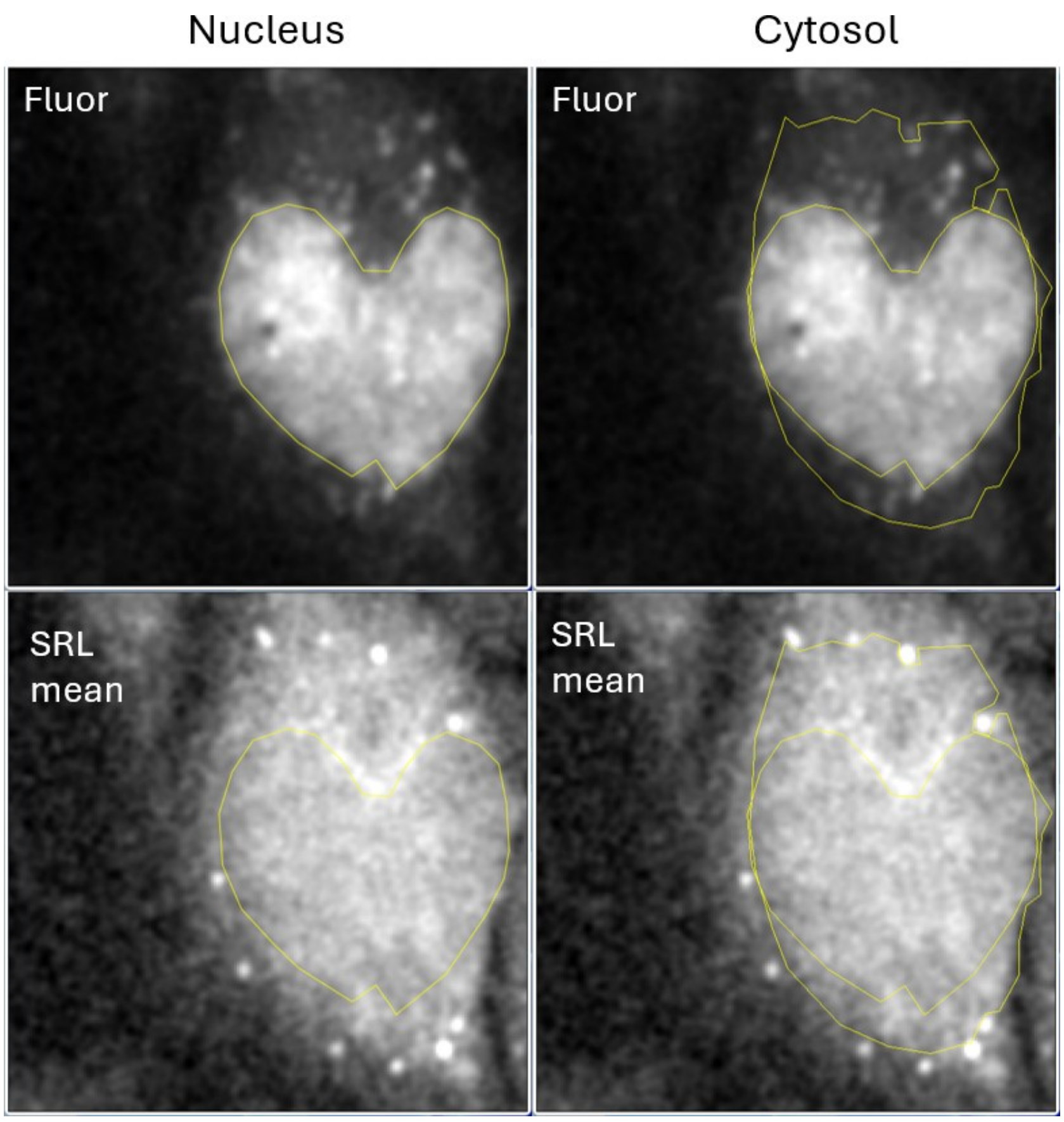

Figure S14. Regions of interest for data shown in Fig.3. Left: fluorescent nuclear region containing mRhubarb720. Right: weakly fluorescent cytosol region used as reference. Data shown taken for IFD 1660 cm^{-1}.

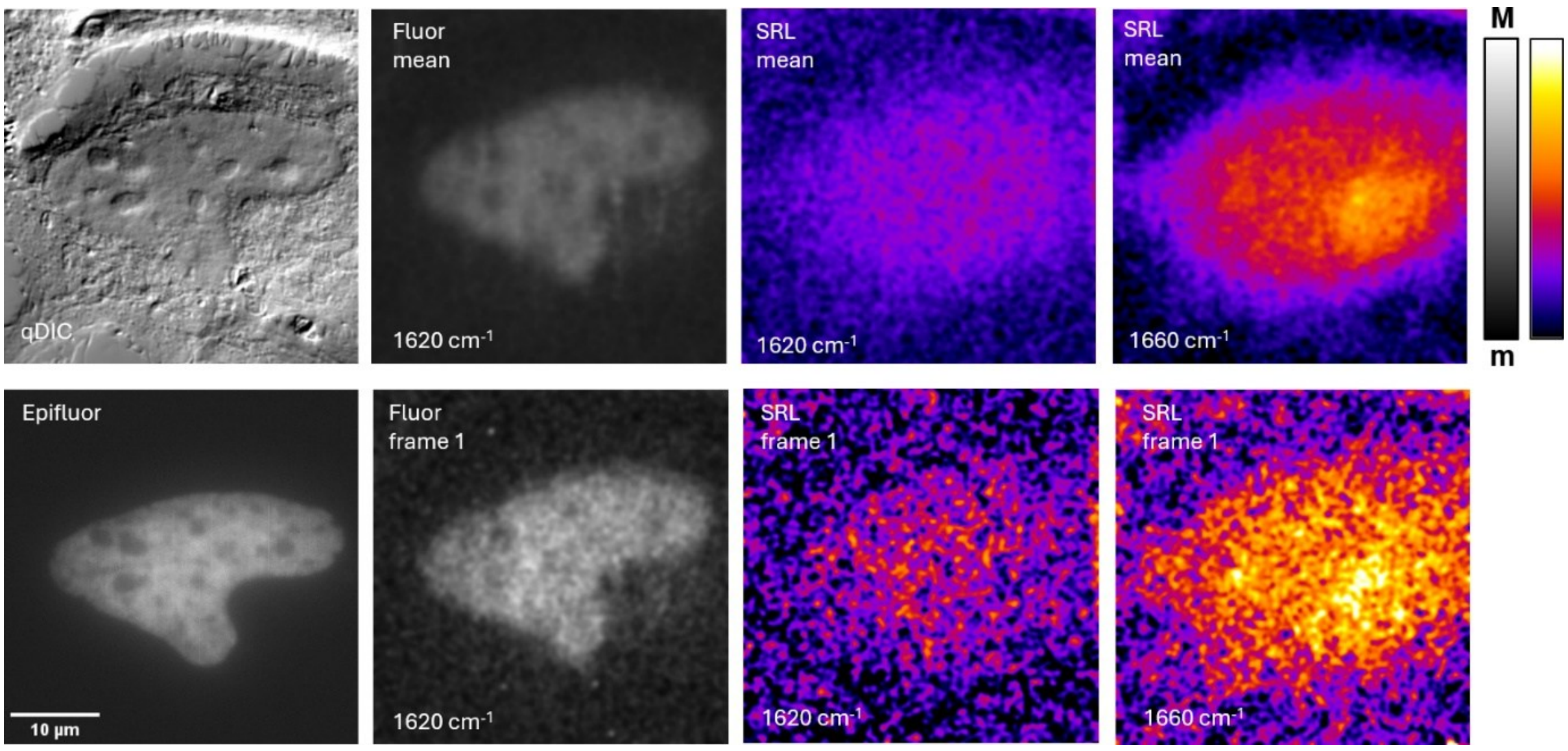

Figure S15: Imaging HeLa cell H2B-labelled mRhubarb720 expression. qDIC, SRL, widefield epi-fluorescence (Epifluor) and forward detected fluorescence (Fluor) are shown, as indicated. Grey and color scales are linear from m to M as follows: qDIC (m = 0.7 rad to M = 0.7 rad),

Epifluor (m = 0 pe to M = 4 kpe, where pe are photoelectrons), Fluor (m = 0 Mpe/s to M = 1 Mpe/s, SRL (m = 0.2 mV to M = 1.3 mV). SRL and Fluor are taken over 40 frames, in alternating between 1620 and 1660 cm^{-1}, 0.01 ms pixel dwell time, 40×40 µm at 371×371 points, about 2s per frame. qDIC and Epifluor is taken before CRS imaging. The mean is taken over the 20 frames using the wavenumber given. Microscope objective 60× 1.27 NA, pump (Stokes) power at the sample 7 mW (12 mW). Centre IFD 1640 cm^{-1}.

Section S8 One and Two-photon absorption and fluorophore symmetry

Epr-SRS requires close to resonant light excitation of a strong dipole-allowed transition (typically via the pump beam, while the Stokes beam is more red-shifted). The transition energy without vibrational excitation can be seen as the reflection symmetry point between emission and absorption spectra (see spectrum in Fig. S17 for Rhodamine 800). As discussed in the main text, red-shifting the light excitation compared to this point reduces the one-photon absorption by the thermal occupation factor. At room temperature, this provides 4 to 5 orders of magnitude reduction for 2000 cm^{-1} shift. The fluorophores used here have a peak molar extinction around ε = 100 mM^{-1} cm^{-1}, corresponding to an absorption cross-section of 3.8×10^{-16} cm^2.

For typical excitation powers of 10 mW at 100 MHz repetition rate at a wavelength of 820 nm focussed to the diffraction limit of a high numerical aperture objective (0.1 μm^2) e.g. our 1.27 NA objective, a peak photon flux of about 4×10^{17} cm^{-2} per pulse is created. Therefore, to have a one-photon absorption probability per pulse well below unity, an absorption cross-section of well below 2.5×10^{-18} cm^2 is required. Considering the detuning of the pump beam at 820 nm from the transition without vibrational excitation around 715 nm for mRhubarb720, a 1790 cm^{-1} shift is estimated, creating a thermal reduction factor of around 5000, which is consistent with the measured absorption (see Fig. S7). For the measured peak extinction coefficient of ε = 72.5 mM^{-1} cm^{-1} (see table S1), the corresponding absorption cross-section is 5×10^{-20} cm^2 at 820 nm, yielding a one-photon absorption probability in the focus per pump pulse of 0.02. This is well below unity, but still is providing a significant excitation, namely an excitation rate of 2 MHz. The corresponding fluorescence is clearly seen in the volume experiment in Fig. S6 comparing non-confocal with confocal detection.

Two-photon absorption is a mechanism which is always relevant as a background for CRS, as it is also a third-order non-linear signal, so that both CRS and TPA scale similarly with excitation pulse parameters and require high-excitation intensities. Assuming 1 ps pulse duration and the above-mentioned pulse parameters, the peak photon flux in the focus is 4×10^{29} $cm^{-2}s^{-1}$. The two-photon cross-sections σ_2 of fluorophores depend strongly on their symmetry, being proportional to the change of the permanent dipole moment between ground and excited state.[26] Typical values of σ_2 are 10-100 GM (1GM=10^{-50} cm^4s), from which we can calculate 0.016 to 0.16 absorbed photons during the 1 ps pulse considered above. Therefore, we find that one and two-photon absorption are on the same order of magnitude here, thus both contributing to photobleaching. Any larger detuning of the pump to reduce the one-photon absorption would reduce the epr-CRS while likely not reducing the excitation of the fluorophore by two-photon absorption. It is thus clear that TPA is in general a significant factor limiting epr-SRS, by providing background signal and creating photobleaching after the excitation. SRS background is created by TPA of the modulated Stokes beam, either by itself,

with the fluorophore creating gain for the pump beam, or as Stokes-pump TPA directly attenuating the pump. The latter is clearly dominating as the background signal has the same sign as SRL. This background is visible in the pump-Stokes delay dependence that we have in spectral focussing (see e.g. Fig. S10) and is discussed in Ref.27 for non-spectral focussing SRS.

TPA can occur at the frequencies $2\nu_p$, $\nu_p+\nu_S$, and $2\nu_S$, which correspond to wavelengths of 410nm, 442nm, and 480 nm, respectively, for the wavelengths of 820 nm (pump) and 960 nm (Stokes) used in our experiment. These wavelengths are shown in Fig. S7, and we see that the pump TPA is spectrally matching with the second absorption band around 400 nm. TPA spectra of iRFP670 and iRFP720 have been reported in Ref.28, although without quantification, as shown in Fig. S16. iRFP720 is spectrally similar to mRhubarb720, with excitation and emission wavelengths equal within 1 nm, and extinction coefficients equal within a few percent (see www.fpbase.org/protein/irfp720/ and www.fpbase.org/protein/mrhubarb720/). However, iRFP720 is a dimer, making it more bulky and thus less suited for genetic labelling. The spectra are consistent with the expectation that the highest TPA is occurring for the pump.

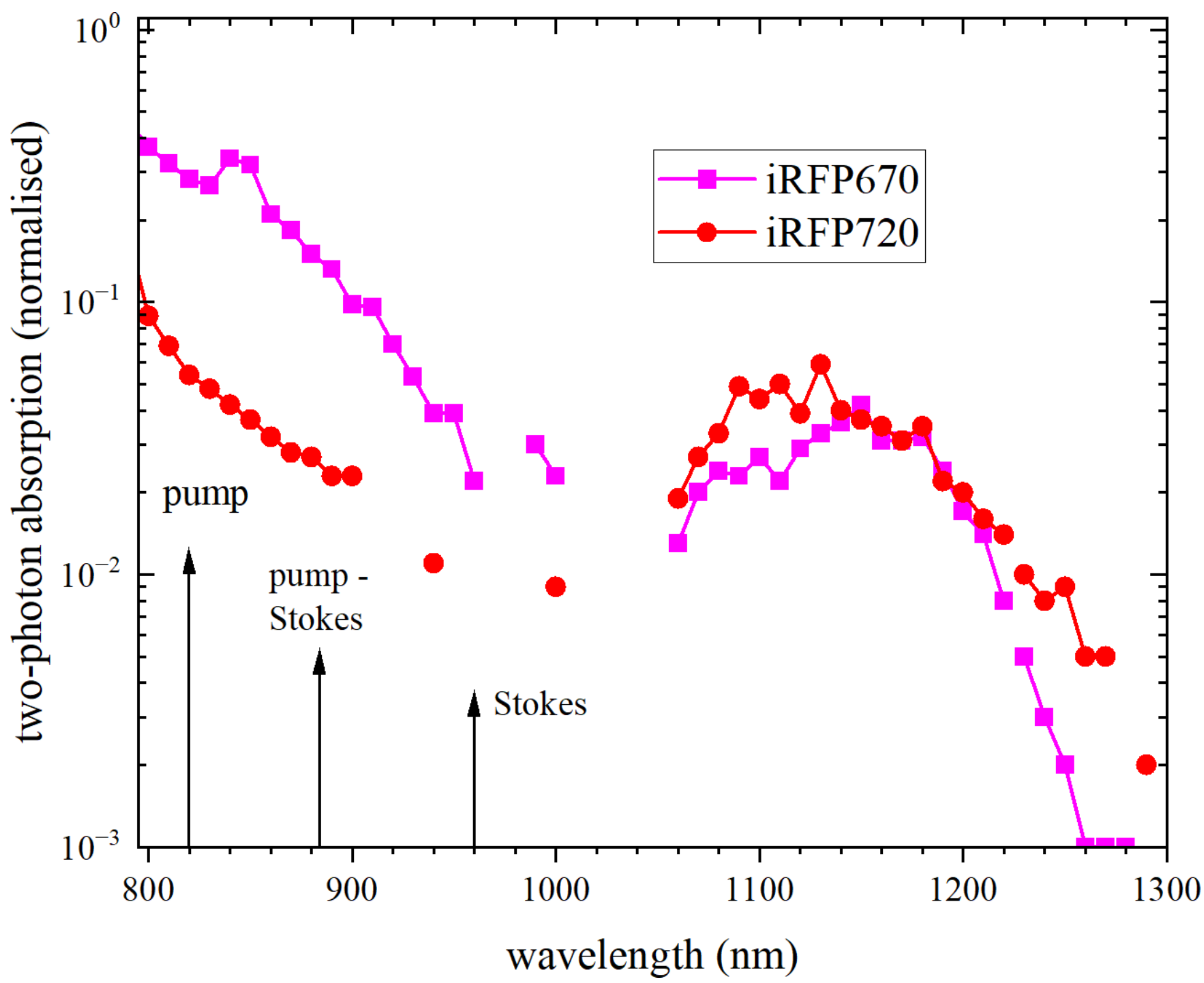

Figure S16. Two-photon absorption of iRFP670 and iRFP720 as reported previously.[28] Data is given normalised in arbitrary units. The pump, Stokes and pump-Stokes wavelength used in the present work are shown as arrows.

In this respect, it is interesting to discuss the SRL signal to background (S/B) ratios for a range of fluorophores reported in Ref.29 (extended data table 1). The S/B ratio is defined as the vibrationally resonant signal relative to the non-resonant signal. The measurements by Wei et al[29] used a Stokes wavelength of 1064 nm and a pump wavelength in the range 860-920 nm, with 6 ps pulses at 80 MHz repetition rate without spectral focussing. In Fig. S17 we show dyes from this data with absorption peaks in the 680 to 710 nm region, selected to approximately retain the detuning from the resonance and thus supress detuning effects on the S/B ratio. Despite this, there are large variations of the reported S/B ratio. Notably, the dyes of high S/B (Rhodamine 800, Atto 700, MARS 2183) have delocalised conjugated bonds of high symmetry and structural rigidity formed by connected rings, while dyes of low S/B have a lower symmetry and conjugated bonds delocalised over a linear chain. This is consistent with the creation of the electronic background by two-photon absorption of the S_0-S_1 transition, which is stronger in less symmetric structures. The spectral shape of the two-photon absorption also plays a role. There are 3 sum frequencies available, $2\nu_p$, $\nu_p + \nu_S$, and $2\nu_S$, which in the reported data correspond to wavelengths of 430-460 nm, 476-494 nm, and 532 nm, respectively. Two-photon absorption spectra of these fluorophores are not available to our knowledge.

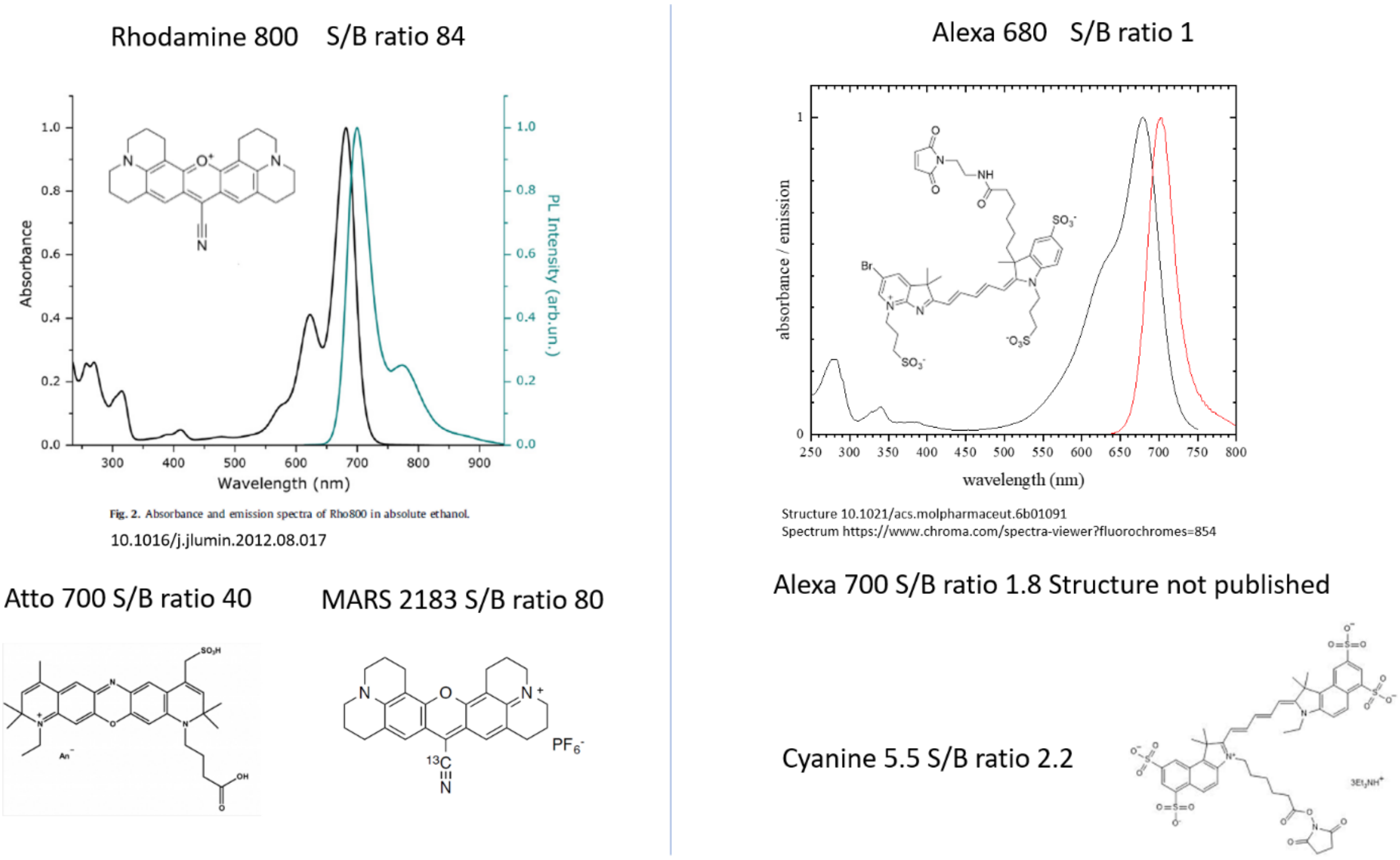

Figure S17. Signal to background (S/B) ratio for selected dyes reported by Wei et al[29]. The S/B ratio is defined as resonant vibrational contrast to non-resonant background in SRL using a Stokes wavelength of 1064nm and a Pump wavelength in the range 860-920nm. The absorption and emission spectra are given for Rhodamine 800 and Alexa 680. All dyes shown have the absorption peak in the 680-710 nm range.

Section S9 Photobleaching during SRS imaging

For the imaging conditions in Fig.3 and S15, with a pixel dwell time of 0.01ms and a pixel size of 108 nm, about a third of the focus size, the exposure time for a given image point (consisting in 2D of 3x3=9 pixels) is about 0.1 ms per frame. The observed bleaching in Fig. 3 appears on a timescale of 10 frames, hence about 1 ms exposure. Assuming an excitation probability of the order of 10% per pulse as estimated in section S8, this corresponds to about 10^4 excitations. We note that typical fluorophores photobleach after some 10^4 to 10^6 excitation cycles for the commonly used continuous wave excitation in their main absorption band. In our pulsed train excitation, there are 800 pulses during the pixel dwell time, which enables excited state absorption by subsequent pulses once an excitation has been created and still present after 12ns when the next pulse arrives. Fluorescence lifetimes of mRhubarb720 are around 1ns, therefore only intersystem crossing into the long-lived triplet state would allow the excitation to be still present when the next pulse arrives. Notably, since the absorption by the triplet state is typically spectrally broad, the absorption of pump and Stokes by this state could be orders of magnitude higher, exaggerating the photobleaching. Using a lower repetition rate laser and/or faster scanning to apply only a few pulses to any given position would enable reduction of such photobleaching.

To investigate the role of long-lived triplet states further, we have measured the bleaching as a function of the scan speed, which is changing the number of consecutive pulses applied to a single mRhubarb720 molecule. Specifically, we repeatedly scanned regions of 40 µm by 30 µm, using 140 horizontal lines spaced vertically by 216 nm, at various scan speeds, and measured the fluorescence bleaching as a function of the total imaging time. To obtain a statistically relevant ensemble for multiple scan speed, a total of 30 regions were imaged, which are shown in Fig. S18. Here, we investigated HeLa cells labelled with the H2B-mRhubarb720 fusion tag, as well as lifeact-mRhubarb720 labelled HeLa cells. The signal and the background were averaged over suited regions as indicated, and their decay over time analysed. In these scans, the IFD was switched between 1620 and 1640 cm^{-1} every 1.4 s, to enable analysis of the SRL signal spectral dependence. As the investigated cells had a lower expression of mRhubarb720, the SRL signal turned out to be too weak for interpretation. We therefore focus here on the fluorescence bleaching.

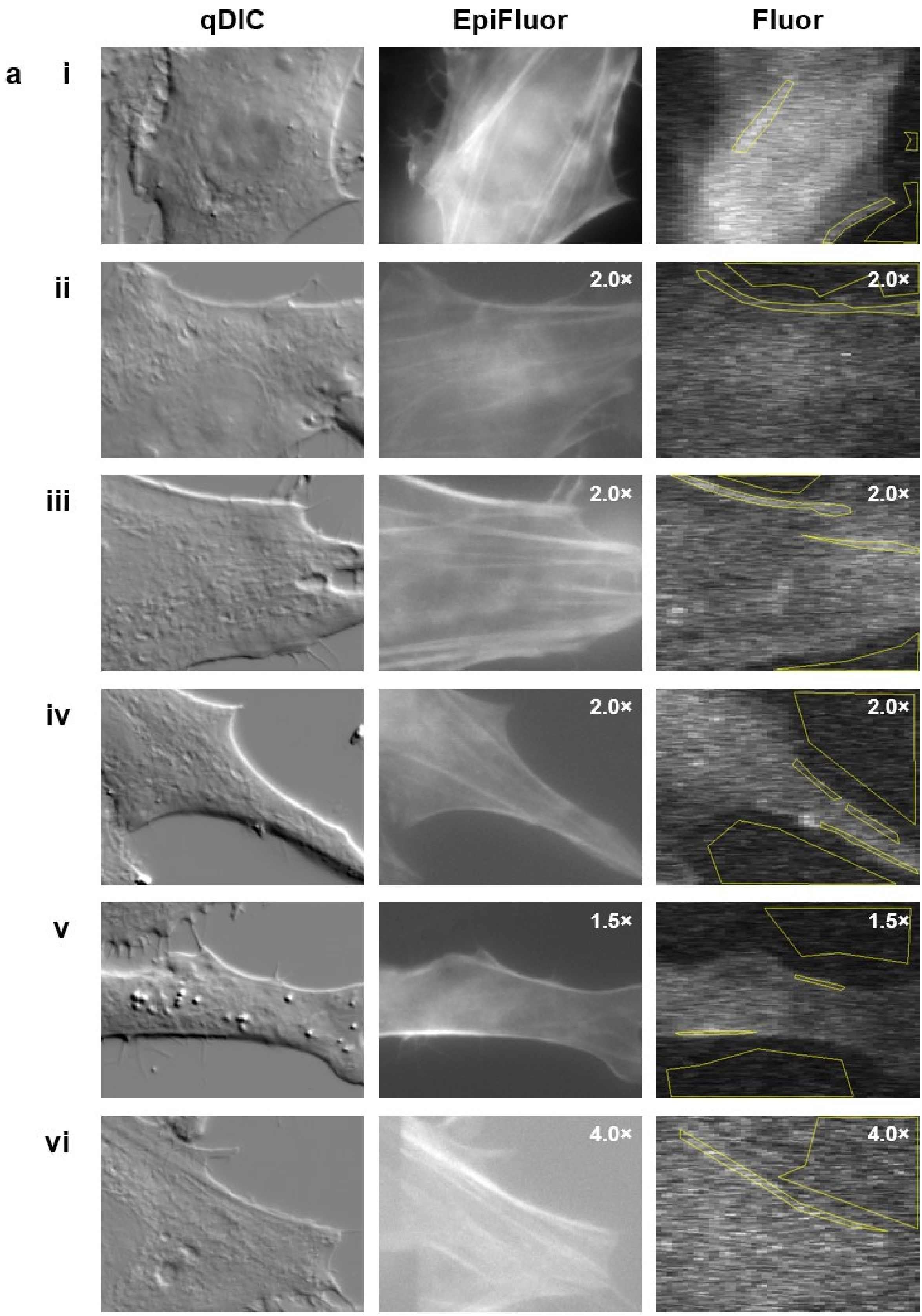
qDIC
EpiFluor
Fluor
a
i
ii
iii
iv
v
vi
2.0×
2.0×
2.0×
2.0×
2.0×
2.0×
1.5×
1.5×
4.0×
4.0×

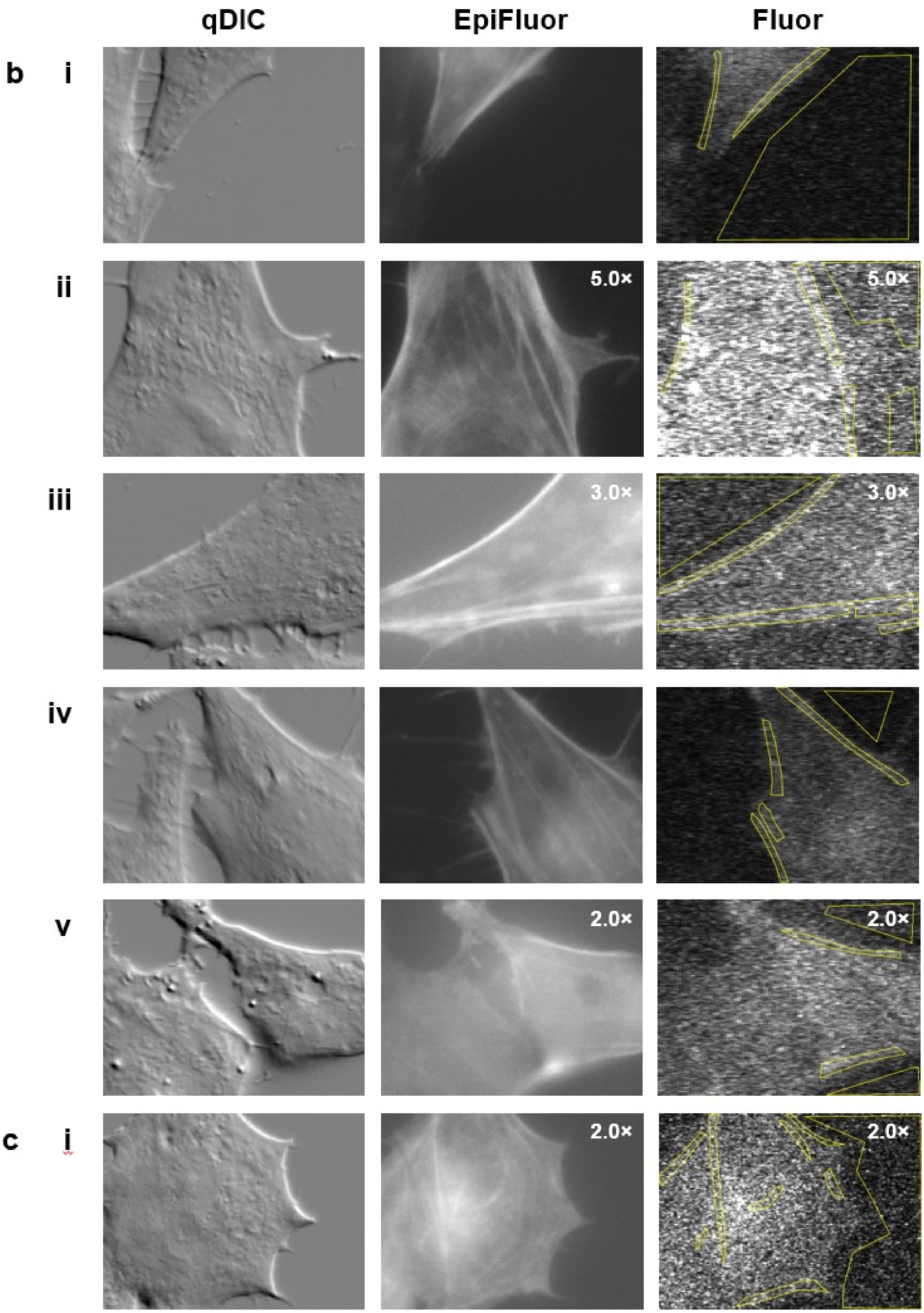

qDIC
EpiFluor
Fluor
b
i
ii
5.0×
5.0×
iii
3.0×
3.0×
iv
v
2.0×
2.0×
c
i
2.0×
2.0×

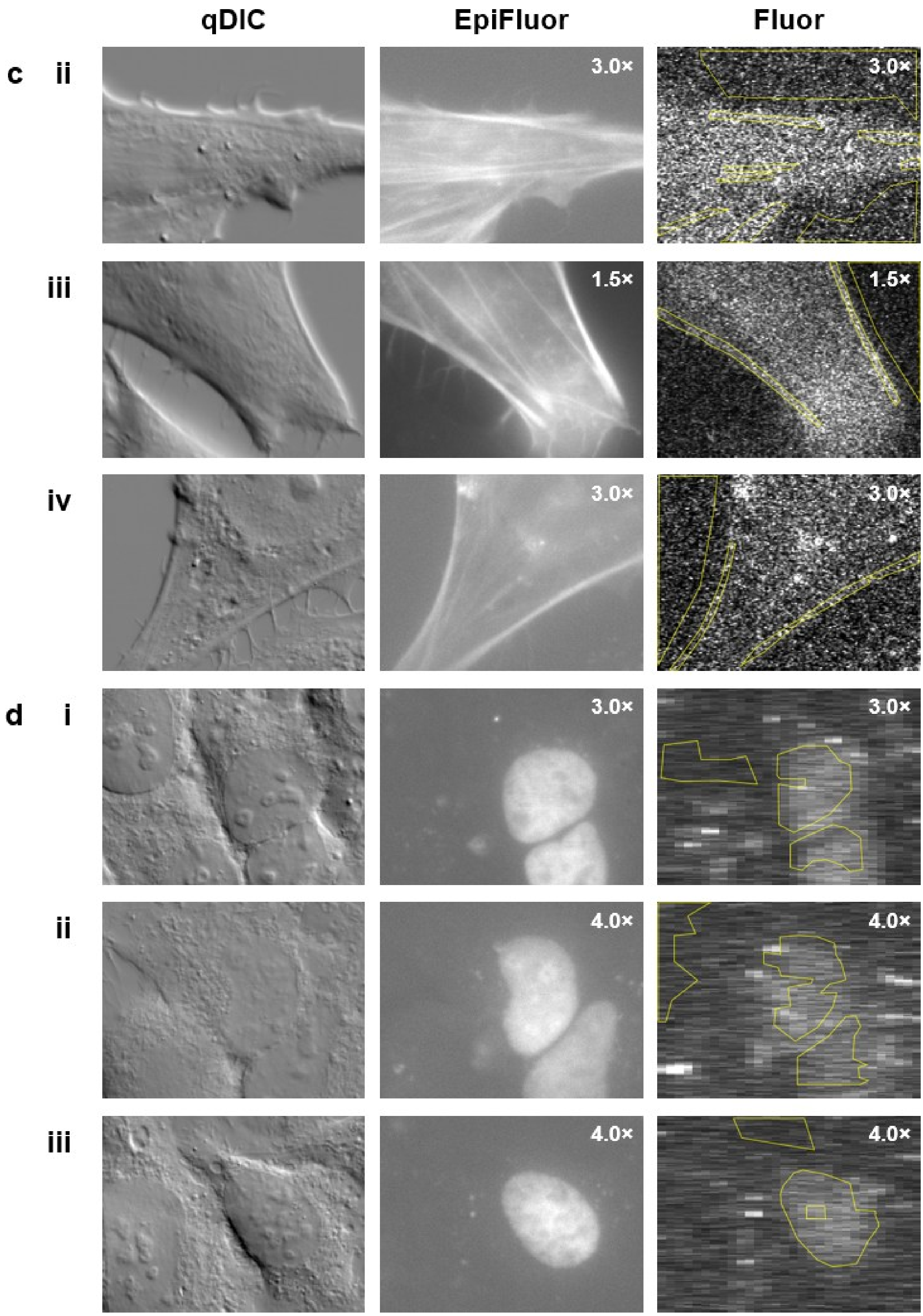
qDIC
EpiFluor
Fluor
c
ii
iii
iv
d
i
ii
iii
3.0×
1.5×
3.0×
3.0×
4.0×
4.0×

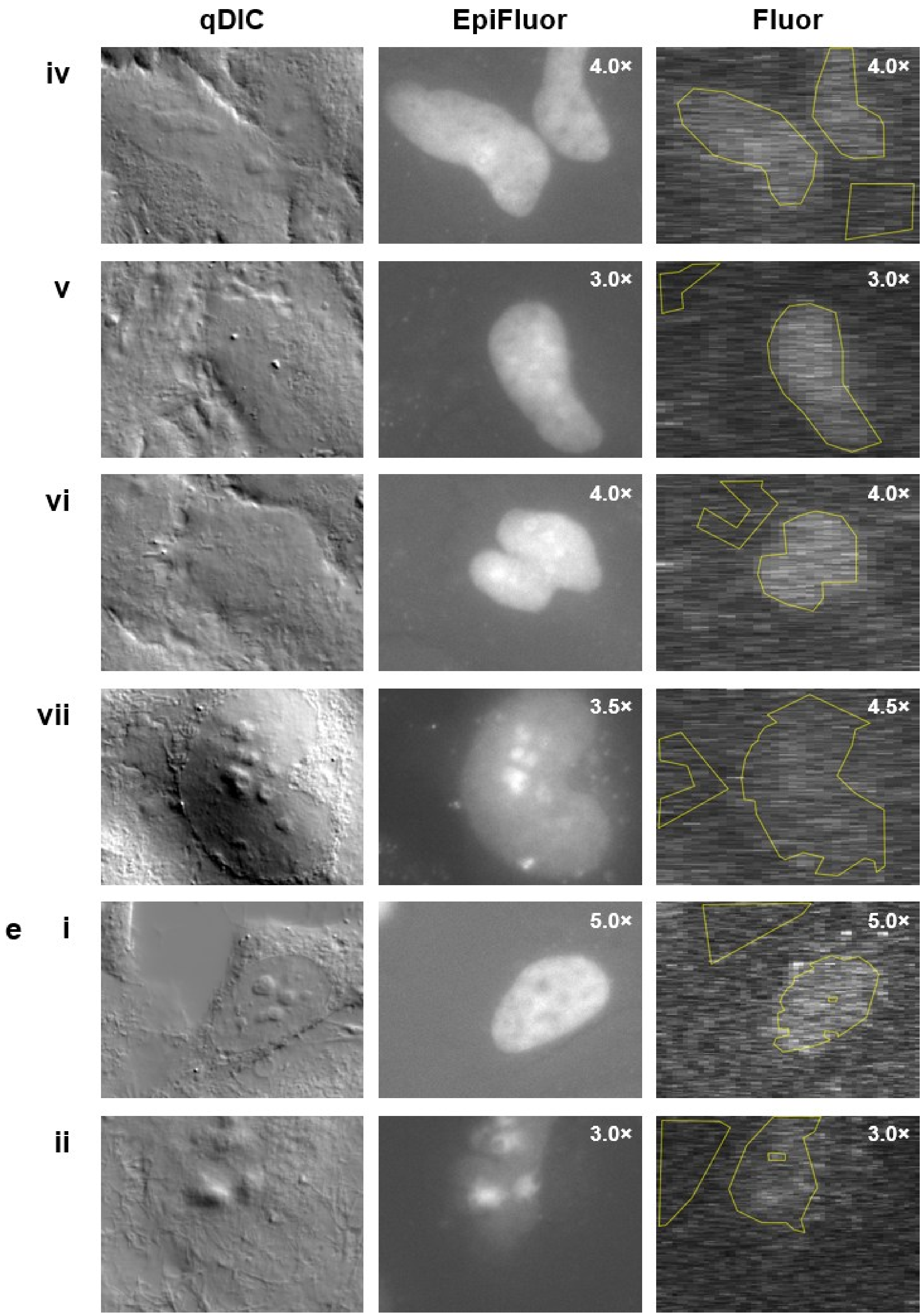
qDIC
EpiFluor
Fluor
iv
4.0×
4.0×
v
3.0×
3.0×
vi
4.0×
4.0×
vii
3.5×
4.5×
e
i
5.0×
5.0×
ii
3.0×
3.0×

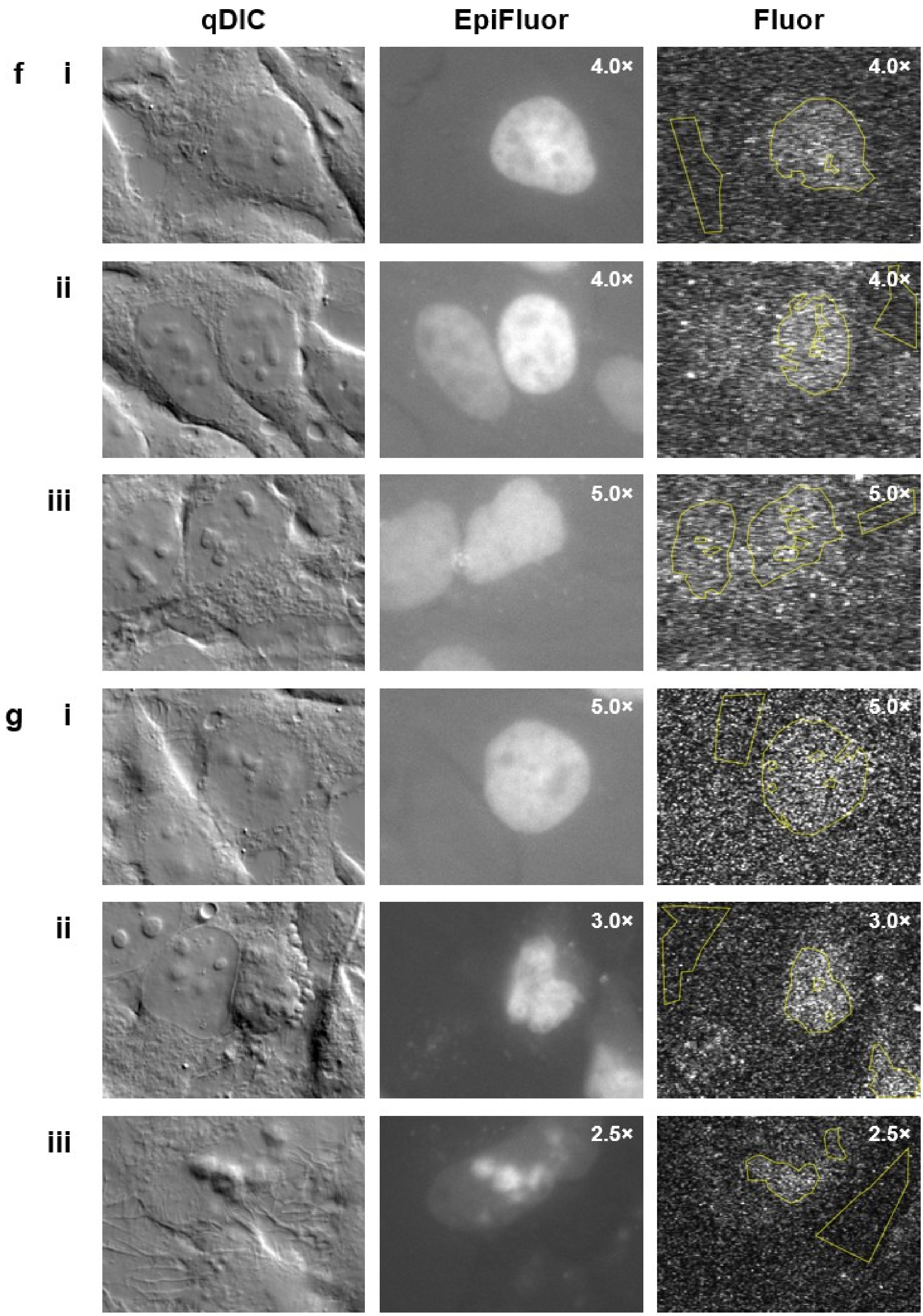

Figure S18. Imaged regions (40 µm by 30 µm) used to study bleaching as function of scan speed. Fixed HeLa cells were stained with lifeact-mRhubarb720 and H2B-mRhubarb720. qDIC (left column), widefield epi-fluorescence (EpiFluor, middle column) and forward detected

fluorescence (Fluor, right column) are shown. Grey scales as black to white as follows: qDIC -0.15 to 0.15 rad (a-c) and -0.20 to 0.20 (d-g); EpiFluor 0 to 5520 pe; Fluor 0 to 850 kpe/s. Data multiplied by factors, as provided. Microscope objective 60× 1.27 NA, pump (Stokes) power at the sample 6 mW (8 mW). Centre IFD 1620 cm^{-1}. The regions used to average the Fluor signal and background are indicated by yellow outlines. The scan speed was adjusted by using different numbers of 10 µs samples across the 40 µm long line. The areas shown are corresponding to a) lifeact, 50 samples; b) lifeact, 100 samples; c) lifeact, 200 samples; d) H2B, 30 samples; e) H2B, 50 samples; f) H2B, 100 samples; g) H2B, 200 samples.

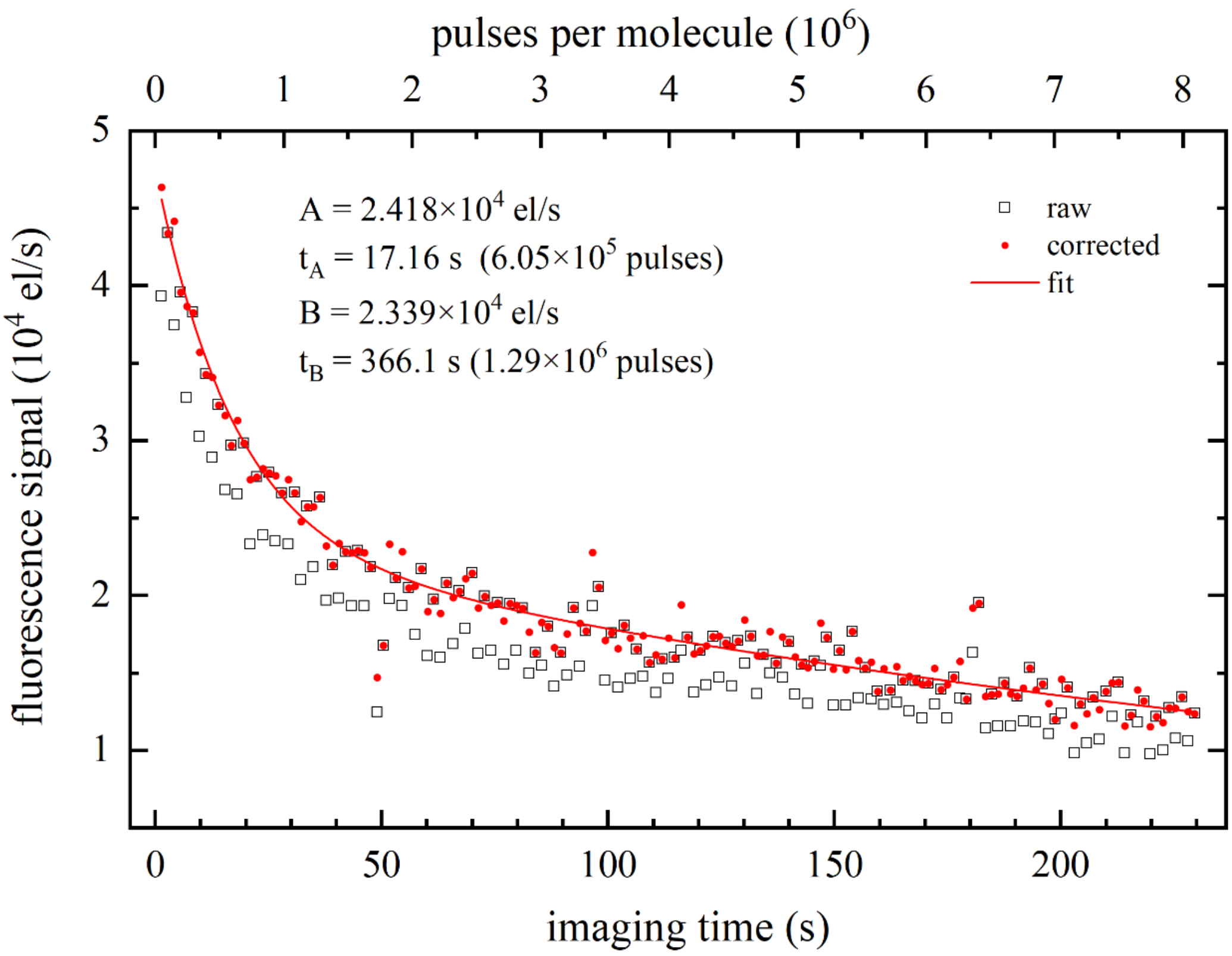

Figure S19. Fluorescence bleaching for a scan of 30 samples (7.5 µs/µm), taken on the region d ii in Fig. S18. The imaging time is converted into accumulated pump pulses per molecule using a PSF area of $(230nm)^2$. Raw measurements (open squares) were corrected (red circles) by applying a factor to the data acquired at 1620 cm^{-1} to compensate for the systematic difference in signal as compared to 1640 cm^{-1}, and then fitted with a double exponential decay (red line) yielding the amplitudes and decay times as shown.

An example of the resulting bleaching curves is shown in Fig. S19. We compensate the small difference, experimentally observed, between the fluorescence signal measured using 1620 cm^{-1} and 1640 cm^{-1} (likely due to the different pulse overlap) by applying a factor of 1.18. The data is fitted by a double exponential decay $Ae^{t/t_A} + Be^{t/t_B}$ with the amplitudes A, B and time constants t_A, t_B shown in Fig. S19. This is done for each of the areas. We note that the total number of pump pulses over the bleaching time is of the order of 10^6, and that the number of Stokes pulses is twice lower due to the 2.5MHz modulation, providing a repeated sequence of 16 pulses on and 16 pulses off.

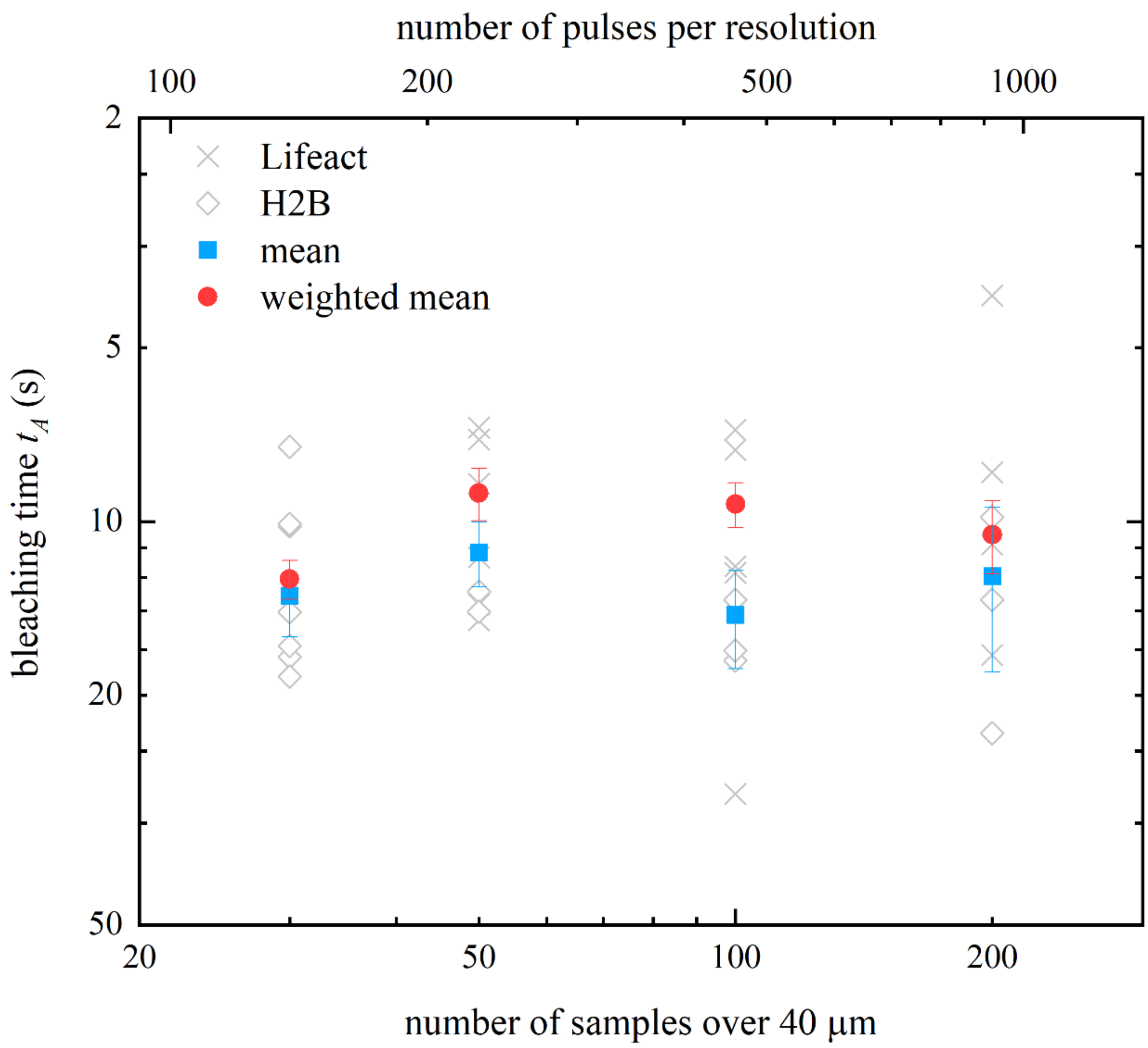

Figure S20. Fluorescence time constants as function of scan speed, given by the number of 10 µs samples across a 40 µm long line, from fits of the data as exemplified in Fig. S19, for all regions shown in Fig. S18. The number of samples is shown on the top axis as number of pulses within the optical resolution of 230 nm. Each region is shown as a symbol, with means at a given number of samples shown as filled squares and weighted means using the fit errors shown as filled circles.

The resulting faster time constant t_A is given in Fig. S20 for all regions shown in Fig. S18. While individual results are fluctuating significantly, there seems to be no overall trend, also when considering the mean or weighted mean. This indicates that triplet-state accumulation is not the dominant bleaching mechanism, as one would expect an accumulation of triplet states leading to excited state absorption over the range of 100 to 1000 pulses shown, which would increase photobleaching. Assuming a 10% excitation probability per pulse and a 10% conversion rate to triplets, triplet state formation should take about 100 pulses. mRhubarb720 triplet lifetimes are expected to be 100-1000 µs, much longer than scan across a resolution.

In summary, this bleaching study as function of the scan speed has shown that in the detected fluorescence, the bleaching is not dominated by triplet state formation or other slow processes such as local heating. Thus, the main bleaching mechanism is likely excited state absorption during a single pulse, initiated by one or two-photon absorption (see discussion in section S8).

An interesting aspect of the pre-resonant excitation is the stimulated emission of an excited fluorophore by the pump and Stokes. The stimulated emission can be expected to be much stronger than the one-photon absorption due to the red-shifted gain spectrum (given by the fluorescence spectrum), as also used in stimulated emission depletion (STED) microscopy.[30,31] Thus, after an excitation, the stimulated emission is expected to remove the excitation efficiently, if there is sufficient time for vibrational relaxation. This could reduce photobleaching. However, as is well known from STED, at the same time, excited state absorption leads to an increase in photobleaching.

Section S10 SRS detection sensitivity

The results shown in Fig.2a at 1640 cm^{-1} centre IFD, after subtracting the TPA contribution (blue solid line), show a peak SRS signal of 5.8 mV. These data were acquired at 1 mM concentration of mRhubarb720, using a pump power of 6 mW and a Stokes power of 15 mW (unmodulated, modulated power is half if this) at the sample, respectively. The DC SRS voltage during these measurements was 79.3 mV. Considering the detector DC transimpedance of 36 Ohm, this corresponds to I = 2.2 mA DC current. The shot-noise in the experiment is calculated as root mean square (rms) current fluctuation given by $\sigma=\sqrt{2qI\Delta f}$ where q is the electron charge and Δf is the frequency bandwidth. With I = 2.2 mA, this corresponds to 26.5 pA/$\sqrt{\mathrm{Hz}}$ and in turn (26.5 pA / 2.2 mA)/$\sqrt{\mathrm{Hz}}$ = 1.2×10^{-8} /$\sqrt{\mathrm{Hz}}$ relative modulation. At 1ms dwell time Δf=500Hz. We detect the SRS current in one quadrature only, which reduces the noise by $\sqrt{2}$, resulting in a relative modulation noise of 1.9×10^{-7}. The signal of 5.8 mV detected for 1 mM concentration, corresponds to a current of 58 nA, using the detector transimpedance of 10^5 Ohm at the SRS resonant modulation (2.5 MHz). Therefore, the detected signal corresponds to a relative modulation of ΔI/I =58 nA / 2.2 mA=2.6×10^{-5}. Scaling the shot-noise limit relative modulation of 1.9×10^{-7} to the value 2.6×10^{-5} at 1 mM, equates to a detection limit concentration of 1 mM × (1.9×10^{-7}) / (2.6×10^{-5}) = 7.2 µM. Previously Wei and Min[32] used 24 mW pump and 120 mW stokes power at the sample, respectively. The detected SRS signal scales linearly with pump and Stokes power, while the noise scales with the square root of the pump power. Therefore, scaling the 7.2 µM concentration limit to these powers results in a detection limit of 7.2 µM × $\sqrt{6/24}$ × (15/120) = 452 nM.

To investigate the concentration dependence and the sensitivity limit experimentally, we have measured a concentration series in solution from 10 µM to 1 mM. The resulting SRL spectra are shown in Fig. S21. The signal is scaling linearly with concentration, and a fit (see inset) provides a scaling constant of 10.2 µV/µM. To estimate the sensitivity, we have determined the noise of these measurements, which was 6.6 µV rms for a dwell time of 0.15 s, corresponding to a detection sensitivity of 0.65 µM. Scaling this value to the 1 ms dwell time used in the above estimate (by multiplying it with $\sqrt{150}$) yields a limit of 8.0 µM, close to the above estimated shot noise limit of 7.2 µM. This confirms close to shot-noise limited detection in our setup, validating the sensitivity estimates above.

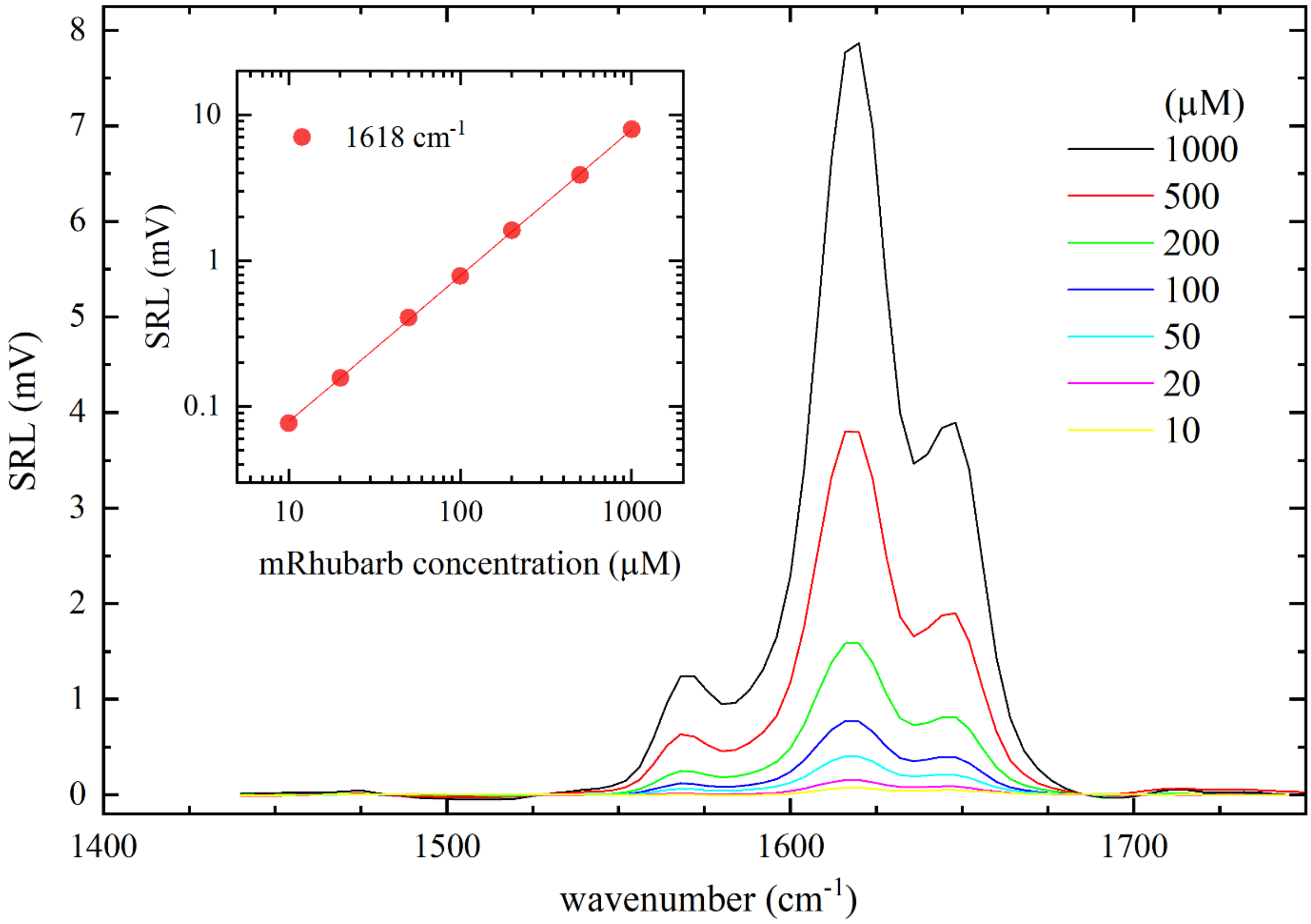

Figure S21. SRL signal of mRhubarb720 in solution at various concentrations from 10 µM to 1 mM as indicated. 6 mW pump and 7.5 mW Stokes power at the sample, 20× 0.75 NA objective, 11 cm^{-1} resolution, centre IFD 1640 cm^{-1}, with TPA background subtracted. The inset shows the peak signal at 1618 cm^{-1} as a function of the concentration with a linear fit yielding a slope of 10.2 µV/µM.

Supporting References

(1) Lambert, T. J. FPbase: A Community-Editable Fluorescent Protein Database. *Nat. Methods* **2019**, *16* (4), 277–278. https://doi.org/10.1038/s41592-019-0352-8.

(2) Rogers, O. C.; Johnson, D. M.; Firnberg, E. MRhubarb: Engineering of Monomeric, Red-Shifted, and Brighter Variants of IRFP Using Structure-Guided Multi-Site Mutagenesis. *Sci. Rep.* **2019**, *9* (1). https://doi.org/10.1038/s41598-019-52123-7.

(3) Matlashov, M. E.; Shcherbakova, D. M.; Alvelid, J.; Baloban, M.; Pennacchietti, F.; Shemetov, A. A.; Testa, I.; Verkhusha, V. V. A Set of Monomeric Near-Infrared Fluorescent Proteins for Multicolor Imaging across Scales. *Nat. Commun.* **2020**, *11* (1). https://doi.org/10.1038/s41467-019-13897-6.

(4) Oliinyk, O. S.; Baloban, M.; Clark, C. L.; Carey, E.; Pletnev, S.; Nimmerjahn, A.; Verkhusha, V. V. Single-Domain near-Infrared Protein Provides a Scaffold for Antigen-Dependent Fluorescent Nanobodies. *Nat. Methods* **2022**, *19* (6), 740–750. https://doi.org/10.1038/s41592-022-01467-6.

(5) Warren, A. J.; Trincao, J.; Crawshaw, A. D.; Beale, E. V.; Duller, G.; Stallwood, A.; Lunnon, M.; Littlewood, R.; Prescott, A.; Foster, A.; Smith, N.; Rehm, G.; Gayadeen, S.; Bloomer, C.; Alianelli, L.; Laundy, D.; Sutter, J.; Cahill, L.; Evans, G. VMXm – A Sub-Micron Focus Macromolecular Crystallography Beamline at Diamond Light Source. *J. Synchrotron Radiat.* **2024**, *31* (6), 1593–1608. https://doi.org/10.1107/S1600577524009160.

(6) Winter, G.; Beilsten-Edmands, J.; Devenish, N.; Gerstel, M.; Gildea, R. J.; McDonagh, D.; Pascal, E.; Waterman, D. G.; Williams, B. H.; Evans, G. DIALS as a Toolkit. *Protein Science* **2022**, *31* (1), 232–250. https://doi.org/10.1002/pro.4224.

(7) Gildea, R. J.; Beilsten-Edmands, J.; Axford, D.; Horrell, S.; Aller, P.; Sandy, J.; Sanchez-Weatherby, J.; Owen, C. D.; Lukacik, P.; Strain-Damerell, C.; Owen, R. L.; Walsh, M. A.; Winter, G. Xia2.Multiplex: A Multi-Crystal Data-Analysis Pipeline. *Acta Crystallogr. D Struct. Biol.* **2022**, *78* (6), 752–769. https://doi.org/10.1107/S2059798322004399.

(8) Jumper, J.; Evans, R.; Pritzel, A.; Green, T.; Figurnov, M.; Ronneberger, O.; Tunyasuvunakool, K.; Bates, R.; Žídek, A.; Potapenko, A.; Bridgland, A.; Meyer, C.; Kohl, S. A. A.; Ballard, A. J.; Cowie, A.; Romera-Paredes, B.; Nikolov, S.; Jain, R.; Adler, J.; Back, T.; Petersen, S.; Reiman, D.; Clancy, E.; Zielinski, M.; Steinegger, M.; Pacholska, M.; Berghammer, T.; Bodenstein, S.; Silver, D.; Vinyals, O.; Senior, A. W.; Kavukcuoglu, K.; Kohli, P.; Hassabis, D. Highly Accurate Protein Structure Prediction with AlphaFold. *Nature 2021 596:7873* **2021**, *596* (7873), 583–589. https://doi.org/10.1038/s41586-021-03819-2.

(9) Collaborative Computational Project, N. 4. The CCP4 Suite: Programs for Protein Crystallography. *Acta Crystallogr. D Biol. Crystallogr.* **1994**, *50* (Pt 5), 760–763. https://doi.org/10.1107/S0907444994003112.

(10) Emsley, P.; Cowtan, K. Coot: Model-Building Tools for Molecular Graphics. *Acta Crystallogr. D Biol. Crystallogr.* **2004**, *60* (Pt 12 Pt 1), 2126–2132. https://doi.org/10.1107/S0907444904019158.

(11) Baloban, M.; Shcherbakova, D. M.; Pletnev, S.; Pletnev, V. Z.; Lagarias, J. C.; Verkhusha, V. V. Designing Brighter Near-Infrared Fluorescent Proteins: Insights from Structural and Biochemical Studies. *Chem. Sci.* **2017**, *8* (6), 4546–4557. https://doi.org/10.1039/C7SC00855D.

(12) Shaner, N. C.; Campbell, R. E.; Steinbach, P. A.; Giepmans, B. N. G.; Palmer, A. E.; Tsien, R. Y. Improved Monomeric Red, Orange and Yellow Fluorescent Proteins Derived from Discosoma Sp. Red Fluorescent Protein. *Nat. Biotechnol.* **2004**, *22* (12), 1567–1572. https://doi.org/10.1038/nbt1037.

(13) Langbein, W.; Regan, D.; Pope, I.; Borri, P. Invited Article: Heterodyne Dual-Polarization Epi-Detected CARS Microscopy for Chemical and Topographic Imaging of Interfaces. *APL Photonics* **2018**, *3* (9). https://doi.org/10.1063/1.5027256.

(14) Langbein, W.; Rocha-Mendoza, I.; Borri, P. Coherent Anti-Stokes Raman Micro-spectroscopy Using Spectral Focusing: Theory and Experiment. *Journal of Raman Spectroscopy* **2009**, *40* (7), 800–808. https://doi.org/10.1002/jrs.2264.

(15) Albrecht, A. C.; Hutley, M. C. On the Dependence of Vibrational Raman Intensity on the Wavelength of Incident Light. *J. Chem. Phys.* **1971**, *55* (9), 4438–4443. https://doi.org/10.1063/1.1676771.

(16) Xiong, H.; Min, W. Combining the Best of Two Worlds: Stimulated Raman Excited Fluorescence. *J. Chem. Phys.* **2020**, *153* (21). https://doi.org/10.1063/5.0030204.

(17) Hamilton, S.; Regan, D.; Payne, L.; Langbein, W.; Borri, P. Sizing Individual Dielectric Nanoparticles with Quantitative Differential Interference Contrast Microscopy. *Analyst* **2022**, *147* (8), 1567–1580. https://doi.org/10.1039/D1AN02009A.

(18) Regan, D.; Williams, J.; Borri, P.; Langbein, W. Lipid Bilayer Thickness Measured by Quantitative DIC Reveals Phase Transitions and Effects of Substrate Hydrophilicity. *Langmuir* **2019**, *35* (43), 13805–13814. https://doi.org/10.1021/acs.langmuir.9b02538.

(19) Frisch, M. J.; Trucks, G. W.; Schlegel, H. B.; Scuseria, G. E.; Robb, M. A.; Cheeseman, J. R.; Scalmani, G.; Barone, V.; Mennucci, B.; Petersson, G. A.; Nakatsuji, H.; Caricato, M.; Li, X.; Hratchian, H. P.; Izmaylov, A. F.; Bloino, J.; Zheng, G.; Sonnenberg, J. L.; Hada, M.; Ehara, M.; Toyota, K.; Fukuda, R.; Hasegawa, J.; Ishida, M.; Nakajima, T.; Honda, Y.; Kitao, O.; Nakai, H.; Vreven, T.; Montgomery, J. A. , Jr.; Peralta, J. E.; Ogliaro, F.; Bearpark, M.; Heyd, J. J.; Brothers, E.; Kudin, K. N.; Staroverov, V. N.; Kobayashi, R.; Normand, J.; Raghavachari, K.; Rendell, A.;

Burant, J. C.; Iyengar, S. S.; Tomasi, J.; Cossi, M.; Rega, N.; Millam, J. M.; Klene, M.; Knox, J. E.; Cross, J. B.; Bakken, V.; Adamo, C.; Jaramillo, J.; Gomperts, R.; Stratmann, R. E.; Yazyev, O.; Austin, A. J.; Cammi, R.; Pomelli, C.; Ochterski, J. W.; Martin, R. L.; Morokuma, K.; Zakrzewski, V. G.; Voth, G. A.; Salvador, P.; Dannenberg, J. J.; Dapprich, S.; Daniels, A. D.; Farkas, Ö.; Foresman, J. B.; Ortiz, J. V.; Cioslowski, J.; Fox, D. J. Gaussian09. Gaussian, Inc: Wallingford CT 2016.

(20) Dennington, R.; Keith, T. A.; Millam, J. M. Gausview5 . Semichem Inc.: Shawnee Mission, KS 2016.

(21) Whitby, F. G.; Phillips, J. D.; Hill, C. P.; McCoubrey, W.; Maines, M. D. Crystal Structure of a Biliverdin IXα Reductase Enzyme–Cofactor Complex. *J. Mol. Biol.* **2002**, *319* (5), 1199–1210. https://doi.org/10.1016/S0022-2836(02)00383-2.

(22) Pascual-ahuir, J. L.; Silla, E.; Tuñon, I. GEPOL: An Improved Description of Molecular Surfaces. III. A New Algorithm for the Computation of a Solvent-excluding Surface. *J. Comput. Chem.* **1994**, *15* (10), 1127–1138. https://doi.org/10.1002/jcc.540151009.

(23) Steindal, A. H.; Ruud, K.; Frediani, L.; Aidas, K.; Kongsted, J. Excitation Energies in Solution: The Fully Polarizable QM/MM/PCM Method. *J. Phys. Chem. B* **2011**, *115* (12), 3027–3037. https://doi.org/10.1021/jp1101913.

(24) Mennucci, B. Polarizable Continuum Model. *WIREs Computational Molecular Science* **2012**, *2* (3), 386–404. https://doi.org/10.1002/wcms.1086.

(25) Sinha, P.; Boesch, S. E.; Gu, C.; Wheeler, R. A.; Wilson, A. K. Harmonic Vibrational Frequencies: Scaling Factors for HF, B3LYP, and MP2 Methods in Combination with Correlation Consistent Basis Sets. *J. Phys. Chem. A* **2004**, *108* (42), 9213–9217. https://doi.org/10.1021/jp048233q.

(26) Drobizhev, M.; Makarov, N. S.; Tillo, S. E.; Hughes, T. E.; Rebane, A. Two-Photon Absorption Properties of Fluorescent Proteins. *Nat. Methods* **2011**, *8* (5), 393–399. https://doi.org/10.1038/nmeth.1596.

(27) Pruccoli, A.; Kocademir, M.; Winterhalder, M. J.; Zumbusch, A. Electronically Preresonant Stimulated Raman Scattering Microscopy of Weakly Fluorescing Chromophores. *J. Phys. Chem. B* **2023**, *127* (27), 6029–6037. https://doi.org/10.1021/acs.jpcb.3c01407.

(28) Leben, R.; Lindquist, R. L.; Hauser, A. E.; Niesner, R.; Rakhymzhan, A. Two-Photon Excitation Spectra of Various Fluorescent Proteins within a Broad Excitation Range. *Int. J. Mol. Sci.* **2022**, *23* (21), 13407. https://doi.org/10.3390/ijms232113407.

(29) Wei, L.; Chen, Z.; Shi, L.; Long, R.; Anzalone, A. V.; Zhang, L.; Hu, F.; Yuste, R.; Cornish, V. W.; Min, W. Super-Multiplex Vibrational Imaging. *Nature* **2017**, *544* (7651), 465–470. https://doi.org/10.1038/nature22051.

(30) Vicidomini, G.; Bianchini, P.; Diaspro, A. STED Super-Resolved Microscopy. *Nat. Methods* **2018**, *15* (3), 173–182. https://doi.org/10.1038/nmeth.4593.

(31) Hell, S. W.; Wichmann, J. Breaking the Diffraction Resolution Limit by Stimulated Emission: Stimulated-Emission-Depletion Fluorescence Microscopy. *Opt. Lett.* **1994**, *19* (11), 780. https://doi.org/10.1364/OL.19.000780.

(32) Wei, L.; Min, W. Electronic Preresonance Stimulated Raman Scattering Microscopy. *J. Phys. Chem. Lett.* **2018**, *9* (15), 4294–4301. https://doi.org/10.1021/acs.jpclett.8b00204.
